# A Novel Polyherbal Formulation Modulates Cyclophosphamide-Induced Cytotoxicity in TM3 Leydig Cells and Delays Fictive Ejaculation in Spinal Cord Transected Male Rats

**DOI:** 10.3390/ph18060803

**Published:** 2025-05-27

**Authors:** Patrick Brice Defo Deeh, Hye-Yong Kim, Kiseok Han, Anbazhagan Sathiyaseelan, Hyun-Jong Cho, Myeong-Hyeon Wang

**Affiliations:** 1 Department of Bio-Health Convergence, Kangwon National University, Chuncheon 24341, Republic of Korea; deehdefo@yahoo.fr (P.B.D.D.); elmo0120@naver.com (K.H.); sathiyaseelan.bio@gmail.com (A.S.); 2 Department of Dental Hygiene, College of Health Science, Kangwon National University, Chuncheon 24341, Republic of Korea; khy0606@kangwon.ac.kr; 3 Department of Pharmacy, College of Pharmacy, Kangwon National University, Chuncheon 24341, Republic of Korea; hjcho@kangwon.ac.kr

**Keywords:** cyclophosphamide, polyherbal formulation, TRPV1, TRPM2, TM3 Leydig cells, fictive ejaculation

## Abstract

**Background**: Cyclophosphamide (CP) chemotherapy is commonly associated with various side effects. The development of an effective therapy capable of counteracting these effects is of great interest. **Objectives**: We evaluated the effects of a novel polyherbal formulation (PHF) on CP cytotoxicity in TM3 cells and fictive ejaculation in rats, and determined its possible mechanism. **Methods**: The phytochemical analysis of PHF was determined by GC-MS. Various oxidative stress-related parameters (DPPH, ABTS^+^, CUPRAC, FRAP, MMP, and DCF-DA) and the cytotoxicity (hemolysis and HET-CAM) of PHF were evaluated. Its effect on fictive ejaculation was tested by recording the electromyographic activities of bulbospongiosus muscles, and the involvement of TRPV1/TRPM2 channels was investigated using their specific agonists and antagonists. **Results**: We found that PHF contained various phytocompounds. PHF prevented CP-induced oxidative stress in TM3 cells, probably due to its strong antioxidant potential. For instance, PHF inhibited apoptosis, lipid peroxidation, and ROS generation. Furthermore, the activities of capsaicin (CAP) and cumene hydroperoxide (CHPx) were significantly lowered by PHF, indicating TRPV1 and TRPM2 inhibition. In the in vivo study conducted in spinal male rats, the number of contractions of the bulbospongiosus muscles was significantly (*p* < 0.001) lowered in the PHF + DOPA (1.54 ± 0.3) and PHF + CAP (2.43 ± 0.74) groups, compared with the DOPA (8.75 ± 0.71) and CAP (7.41 ± 1.01) groups, respectively. Additionally, PHF delayed the pro-ejaculatory effects of dopamine (by 17.6%) and capsaicin (by 32.69%). The in silico study revealed a strong binding affinity between the selected PHF phytocompounds and the active pockets of TRPV1 and TRPM2. HET-CAM and hemolysis assays revealed no harmful effects of PHF. **Conclusions**: PHF prevented CP cytotoxicity in TM3 cells and delayed the pro-ejaculatory effects of dopamine and capsaicin in spinal rats through dopamine and TRPV1 inhibition. PHF could be a potential candidate for the management of CP chemotherapy-related disorders, such as premature ejaculation, in particular.

## 1. Introduction

**Figure 1:**
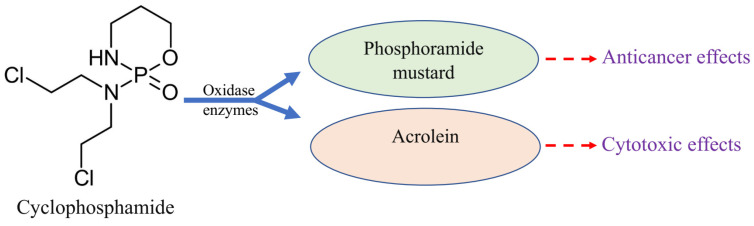
Simple representation of cyclophosphamide metabolism in the liver.

Cyclophosphamide (CP) is one of the main chemotherapy drugs used in the treatment of various cancers, including breast, colon, prostate, ovarian, and testicular cancers [1]. After administration, CP is metabolized into its active forms, phosphoramide mustard and acrolein, by the action of oxidase enzymes in the liver (Figure 1). The anticancer properties of CP are attributed to phosphoramide mustard, while its toxic effects are mediated by acrolein [2]. Acrolein disrupts the antioxidant defense mechanisms in tissues, leading to reactive oxygen species (ROS) overproduction, which may cause oxidative stress and cytotoxicity [3]. The cytotoxic effects of CP particularly target rapidly proliferating cells; hence, the testicular cells are one of the main targets of the destructive effects of this drug [4].

Transient receptor potential vanilloid 1 (TRPV1) and transient receptor potential melastatin 2 (TRPM2) are non-selective ion channels that, in response to a stimulus, induce an inward current of cations, mainly calcium and sodium, which results in depolarization of the cell [5]. Both channels are well expressed in TM3 cells and are involved in the regulation of various physiological processes such as oxidative stress, apoptosis, mitochondrial membrane stability, and cell migration [5,6]. Capsaicin (CAP) and ADP-ribose are TRPV1 and TRPM2 activators, respectively, while Capsazepine (CPZ) and N-(p-amylcinnamoyl) anthranilic acid (ACA) are TRPV1 and TRPM2 blockers, respectively [7]. Among testicular cells, Leydig cells are the main cells involved in sexual response through testosterone, known as a pro-sexual agent [8]. In the present study, TM3 Leydig cells were selected as studies show that TRPV1 and TRPM2 channels are well expressed in these cells [5,6]. The molecular mechanism by which CP induces ejaculatory dysfunction is not clear. However, it could be related to oxidative stress that affects testicular function and alters the production of ejaculatory markers such as dopamine, oxytocin, and serotonin [9,10].

The possibility of using an adjuvant therapy that could prevent the detrimental effects of CP on healthy cells, including Leydig cells, is of great interest. In the present study, we focused on a novel polyherbal formulation (PHF) made with the mixture of five pharmacologically important plants, i.e., *Mondia whitei*, *Dracaena arborea*, *Bridelia ferruginea*, *Guibourtia tessmannii*, and *Helichrysum odoratissimum*, based on their traditional uses and previous experimental studies. *M. whitei* is an aromatic plant commonly called “Limte” in the West Region of Cameroon. Its leaves are used as vegetables, while its roots are consumed as spices or remedies for the treatment of various diseases like erectile dysfunction and premature ejaculation [11]. We have previously reported that this plant improves sexual performance by acting on the spinal generator of ejaculation [12,13]. *D. arborea* is a tall tree commonly called “keubgouh” in Cameroon. Its roots are mixed with palm wine and used as a sexual booster. The pro-sexual and antioxidant activities of this plant have been reported [14]. *B. ferruginea* is a tropical plant traditionally called “Kimi” in Hausa (North Cameroon), and it is used in the treatment of various diseases, including infertility and erectile dysfunction [15]. Our research team reported the pro-sexual effects of this plant in sexually naive male rats [16], as well as its antioxidant activity in PC3, NIH3T3, and BT474 cells [17]. *G. tessmannii* is a large tree known as “Essingang” in Cameroon and commonly used as a sexual stimulant by the indigenous population. We demonstrated in our previous studies that this plant activates the ejaculatory motor pattern of ejaculation via D1 and D2 dopaminergic receptors [18], and modulates oxidative stress in R2C tumor Leydig cells through TRPV1 channels [19]. *H. odoratissimum*, commonly called ‘‘Mbantchuet” in Cameroon, is an aromatic plant used to treat various ailments such as cancers and diabetes. This plant can protect the male reproductive system from the adverse effects of CP, possibly by acting as an antioxidant and increasing the expression of Ckit genes [20]. The mixture of these plants is used by Cameroonian traditional healers despite the lack of scientific and experimental evidence. To our knowledge, no previous studies have described the activities of PHF on CP-induced cytotoxicity. With the hypothesis that the antioxidant compounds present in PHF could protect Leydig cells against the detrimental effect of CP, the present study was conducted to investigate the beneficial effects of PHF on CP-induced TM3 cell cytotoxicity, and to determine its effect on fictive ejaculation in spinal cord transected rats. This was done by evaluating various biological parameters such as oxidative stress-related parameters (DPPH, ABTS, Cupric and Ferric reducing power, and ROS generation), cell viability, apoptosis, mitochondrial membrane potential, and cell migration. The cytotoxicity study of PHF was also determined by a hemolysis assay using human red blood cells, an ex vivo HET-CAM irritation toxicity assay using fertilized eggs, and cellular morphological changes detected microscopically. Since CP chemotherapy is associated with ejaculatory dysfunctions in men, we further evaluated the effects of PHF on fictive ejaculation and determined its possible mechanism of action. The fictive ejaculation model is a reliable method to investigate the effects of drugs on the ejaculatory motor pattern by recording the electromyographic activities of bulbospongiosus muscles and the expression of intra-seminal pressure in anesthetized animals such as male rats [21,22,23].

## 2. Results and Discussion

### 2.1. GC-MS Analysis and TP and TF Contents in PHF

**Figure 2:**
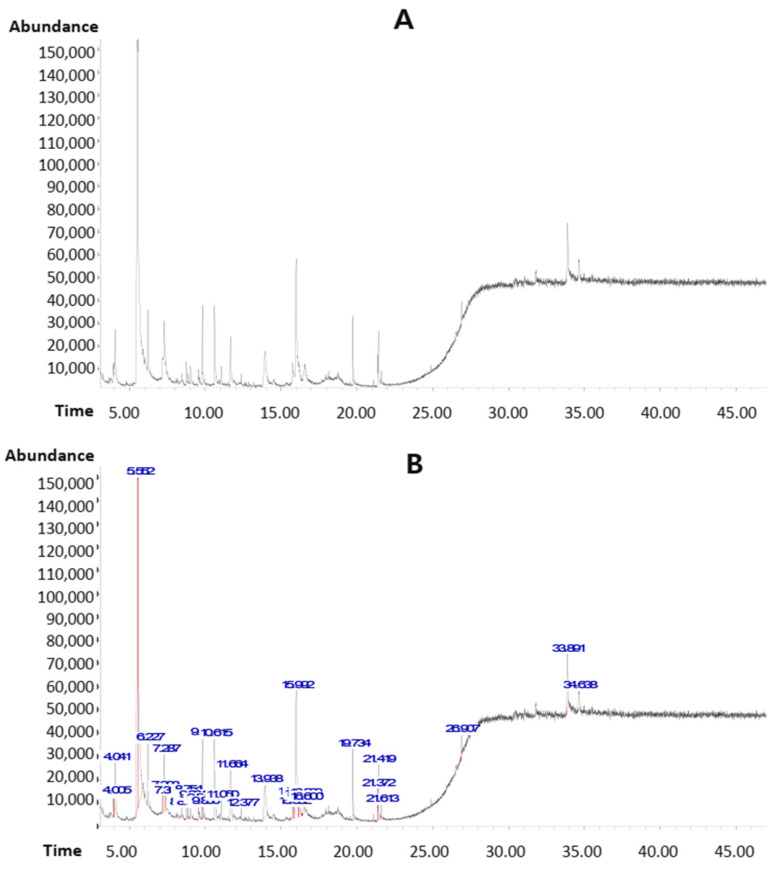
GC-MS analysis of PHF. (**A**,**B**): Chromatograms showing major phytocompounds identified in PHF. The list of various phytocompounds detected in PHF is presented in Table 1.

In this study, a novel polyherbal formulation (PHF) made with the mixture of five plants (*Mondia whitei*, *Dracaena arborea*, *Bridelia ferruginea*, *Guibourtia tessmannii*, and *Helichrysum odoratissimum*) was prepared (20% of each plant), based on their traditional uses and previous findings. The phytocompounds detected in PHF by GC-MS are summarized in Figure 2 and Table 1. Various phytocompounds of pharmacological importance were detected. The pharmacological benefits of some compounds detected in PHF are known. For instance, hydroquinone is known as a compound with potent antimicrobial, anti-inflammatory, skin-whitening, anticancer, pro-apoptotic, and antioxidant potentials. Previous in vivo studies revealed the anorexic and anti-autistic activities of norephedrine. Various pharmacological properties of erythritol, quinic acid, palmitic acid, eicosadienoic acid, linoleic acid, and oleic acid have been documented (Table S1). On the other hand, the TP and TF contents in PHF were 150.88 ± 6.51 mg of GAE/g DW and 5.63 ± 0.71 mg of QE/g DW, respectively (Table S2). The high TP and TF contents and the presence of various phytocompounds of pharmacological importance may justify the use of PHF by traditional practitioners.

### 2.2. Antioxidant Activity of PHF

**Figure 3:**
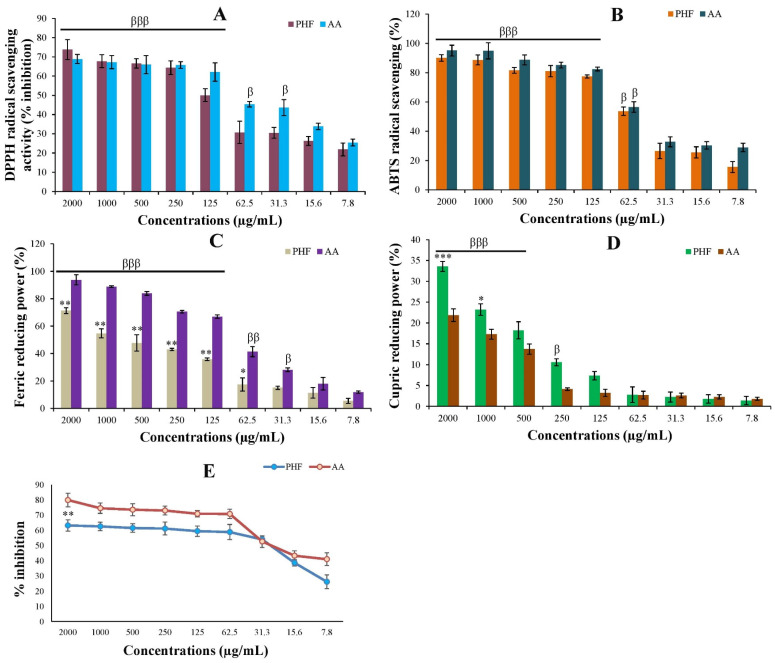
In vitro antioxidant potential of PHF. DPPH (**A**) and ABTS (**B**) free radical scavenging activity, FRAP (**C**) and CUPRAC (**D**) values, and inhibition of lipid peroxidation (**E**). For all parameters, the antioxidant activity of PHF and ascorbic acid was concentration-dependent. Each bar represents the mean ± SEM. Data represent mean ± standard error of the mean (SEM). * *p* < 0.05; ** *p* < 0.01; *** *p* < 0.001: compared to AA. ^β^ *p* < 0.05; ^ββ^ *p* < 0.01; ^βββ^ *p* < 0.001: compared to dose 7.8 µg/mL.

As shown in Figure 3 and Table 2, the DPPH, ABTS^+^, CUPRAC, and FRAP values of PHF and AA were dose-dependent. Indeed, the DPPH radical scavenging activity of PHF at high doses (500–2000 µg/mL) was slightly (*p* > 0.05) higher than that of AA, while AA exhibited better activity at low or moderate doses (7.8–250 µg/mL) (Figure 3A). PHF also displayed a strong ABTS^+^ radical scavenging potential at all doses, but the activity was slightly lower (*p* > 0.05) than that of AA (Figure 3B). Similarly, at doses of 62.5–2000 µg/mL, the FRAP activity of AA was significantly (*p* < 0.05–0.01) higher than that of PHF. However, the FRAP activity of PHF at a dose of 2000 µg/mL was high (above 70%) (Figure 3C). Interestingly, at doses of 1000–2000 µg/mL, we noticed that PHF exhibited a significant (*p* < 0.05–0.001) increase in CUPRAC activity, compared to AA (Figure 3D).

The lipid peroxidation (LP) inhibitory activity of PHF was also tested using egg yolk homogenate as a lipid-rich medium. The results showed that PHF and AA strongly inhibited LP in a concentration-dependent manner (Figure 3E). For instance, at a dose of 2000 µg/mL, PHF and AA inhibited LP by 63.27% and 80.01%, respectively. PHF (2000 µg/mL) showed the highest DPPH (73.70%), ABTS^+^ (95.12%), and CUPRAC (33.56%) activities, while AA exhibited the highest FRAP (93.79%) and LP (80.01%) activities. PHF is a polyherbal formulation fabricated with a mixture of five tropical plants with proven antioxidant potential. Indeed, according to our recent report, *M. whitei* and *G. tessmannii* exhibit a potent antioxidant effect against H_2_O_2_-induced oxidative stress in PC3 cells, with *G. tessmannii* being the most potent [17]. Similarly, studies have reported the antioxidant effect of *D. arborea* [14] and *B. ferruginea* [24] in vivo in adult rats, and *H. odoratissimum* [25] in vitro in A549 lung cancer cells. Thus, the antioxidant effect of PHF observed in the present study is evident. The antioxidant potential of PHF could be attributed to its content of antioxidant compounds. For instance, the antioxidant effects of hydroquinone, erythritol, quinic acid, linoleic acid, and oleic acid have been reported (Table S1).

### 2.3. Protective Effects of PHF on the Detrimental Effects of CP in TM3 Cells

#### 2.3.1. Cell Viability of TM3 Cells

**Figure 4:**
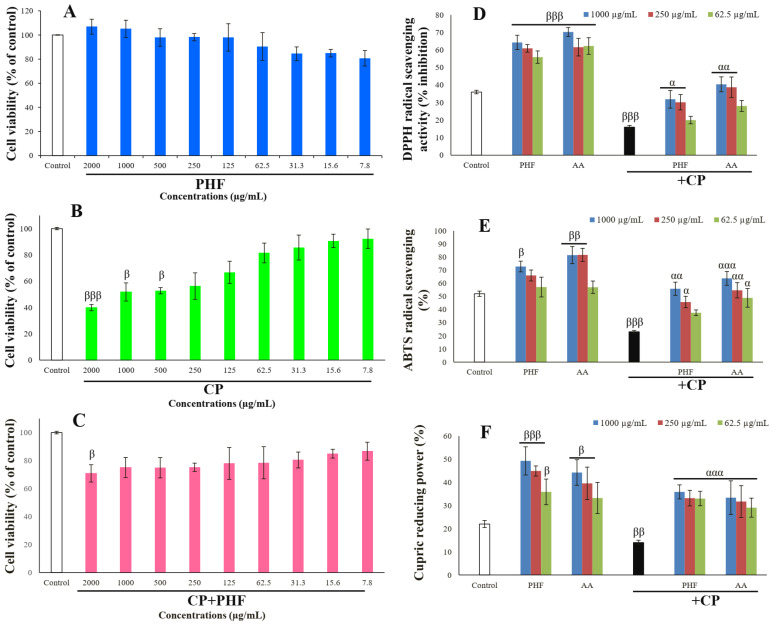
Effects of PHF and/or CP on TM3 cell viability (**A**–**C**), and antioxidant status in cultured cells (**D**–**F**). A trend of increase in cell viability was observed in the cells treated with the highest dose (2000 µg/mL) of PHF, while CP decreased the viability of TM3 cells at high or moderate doses (125–2000 µg/mL). PHF also prevented CP-induced oxidative stress in TM3 cells. Each bar represents the mean ± SEM. Data represent mean ± standard error of the mean (SEM). ^β^ *p* < 0.05; ^ββ^ *p* < 0.01; ^βββ^ *p* < 0.001: compared to negative control; ^α^ *p* < 0.05; ^αα^ *p* < 0.01; ^ααα^ *p* < 0.001: compared to CP.

The effects of CP, PHF, and their combination on the viability of TM3 cells after 24 h of incubation are presented in Figure 4A–C. There were no significant changes in the cell viability after exposure to various concentrations of PHF. However, an increasing trend was observed at high doses (1000–2000 µg/mL), indicating that PHF may contain compounds capable of nourishing cells (Figure 4A). For example, among the compounds detected in PHF, oleic acid has been reported to improve the viability of 786-O cells by activating the GPR40/ILK/Akt pathway. Linoleic acid stimulates the viability of T47D cells and promotes cell growth in a concentration-dependent fashion. Moreover, norephedrine improves PDAC cell viability by acting on the Notch-1 pathway, while erythritol increases HUVEC-CRL-1730 cell viability under hyperglycemic conditions. In this study, CP significantly (*p* < 0.05–0.001) decreased TM3 cell viability at high to moderate doses (500–2000 µg/mL), compared to the control (Figure 4B). This result follows the study of Lee and Kang [26], who reported a significant (*p* < 0.05) decrease in the viability of IPEC-J2 cells treated for 24 h with CP (1–2 µM). Interestingly, PHF enhanced the viability of TM3 cells exposed to CP, compared to CP alone, likely due to the presence of compounds like oleic acid, linoleic acid, and norephedrine (Figure 4C). Thus, PHF could be a potential pro-proliferative agent.

#### 2.3.2. Antioxidant Activities of PHF in TM3 Cells

In the current study, the effect of PHF on CP-induced oxidative stress in TM3 cells was also investigated. PHF and AA (62.5, 250, and 1000 µg/mL) significantly (*p* < 0.05–0.001) increased the DPPH and ABTS free radical scavenging potential, as well as the CUPRAC value in cultured cells, compared to the control (Figure 4D,F). On the contrary, CP significantly (*p* < 0.01–0.001) decreased these parameters. However, PHF significantly (*p* < 0.01–0.001) improved the antioxidant parameters in the presence of CP. Since the cytotoxic effects of CP in patients undergoing long-term chemotherapy particularly target rapidly proliferating cells such as Leydig cells [4], PHF could be a potential candidate for preventing the adverse effects of CP on reproductive function.

#### 2.3.3. Apoptosis Through TRPV1 and TRPM2 Activation in TM3 Cells

**Figure 5:**
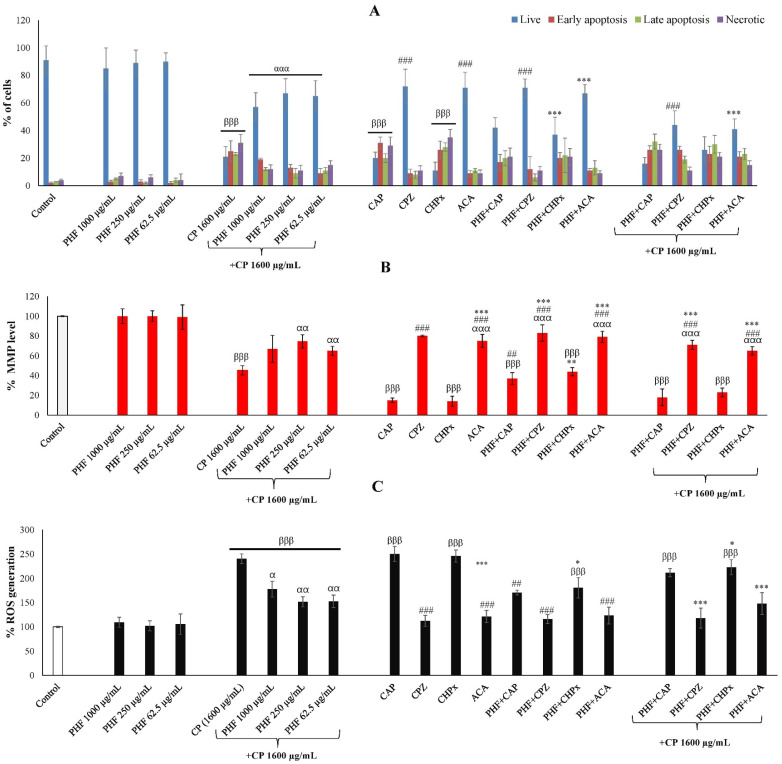
Effects of PHF and CP on apoptosis (**A**), mitochondrial membrane potential (**B**), and ROS generation (**C**) in TM3 cells through TRPV1 and TRPM2 activation. The cells were incubated for 24 h with PHF in the absence or presence of CP and/or TRPV1 and TRPM2 agonists and antagonists. The apoptotic cells, mitochondrial membrane potential, and ROS generation were detected by AO/EB, rhodamine-123, and DCF-DA, respectively. Each bar represents the mean ± SEM. PHF: polyherbal formulation; CP: cyclophosphamide (1600 µg/mL); CAP: capsaicin (0.01 mM); CPZ: Capsazepine (0.1 mM); CHPx: cumene hydroperoxide (1 mM); ACA: N-(p-amylcinnamoyl)anthranilic acid (25 μM). ^βββ^ *p *< 0.001: compared to control; ^α^ *p *< 0.05; ^αα^ *p *< 0.01; ^ααα^ *p *< 0.001: compared to CP; ^##^ *p *< 0.01; ^###^ *p *< 0.001: compared to CAP; * *p *< 0.05; ** *p *< 0.01; *** *p *< 0.001: compared to CHPx.

CP is an effective anticancer drug that can induce high apoptosis in healthy and cancer cells [27,28]. The development of an effective and safe therapy capable of preventing its harmful effects on healthy cells is of great interest. The TM3 cells were incubated for 24 h with PHF (62.5, 250, and 1000 µg/mL) in the absence or presence of CP (1600 µg/mL) and agonists or antagonists of TRPV1 and TRPM2, and apoptosis was determined by AO/EB staining. There were no significant differences in the percentage of live cells, early apoptotic cells, late apoptotic cells, and necrotic cells between the PHF groups and the control group (Figure 5A and Figure S1). In all these groups, the percentage of live cells exceeded 80%. In contrast, we found that CP induced apoptosis in TM3 cells, characterized by a significant increase (*p* < 0.001) in apoptotic and necrotic cells, with a significant (*p* < 0.001) decline in live cells (compared to control). Interestingly, PHF prevented the pro-apoptotic effect of CP, with the highest activity observed at a dose of 250 µg/mL. Activation of TRPV1 [29,30] and TRPM2 [31] channels has been reported to induce apoptosis in various cell lines. To investigate the involvement of TRPV1 and TRPM2 channels in PHF-attenuated TM3 Leydig cell apoptosis, the cells were co-treated with known agonists or antagonists of these channels. We found that CAP (a TRPV1 agonist) and CHPx (a TRPM2 agonist) significantly (*p* < 0.001) increased the percentage of apoptotic cells, while CPZ and ACA tended to decrease apoptosis. These results are consistent with previous reports that showed that CAP promoted apoptosis in various cancers and healthy cells. For instance, CAP stimulates apoptosis in colon cancer cells via ROS overproduction and impairment of mitochondrial transmembrane integrity, as well as in melanoma cells OS (healthy cells) by activating caspase cascades [32]. Similarly, CHPx promotes apoptosis in various cell lines, such as CCL-97 Leydig cells [33] and PC12 cells [34]. In the present study, we found that PHF strongly inhibited the pro-apoptotic effects of CAP and CHPx (Figure 5A and Figure S1). Thus, the anti-apoptotic effect of PHF in TM3 cells could be mainly mediated through TRPV1 and TRPM2 inhibition.

#### 2.3.4. Mitochondrial Membrane Potential (MMP) Through TRPV1 and TRPM2 Activation in TM3 Cells

There were no significant differences in the MMP% between the cells incubated with PHF (62.5, 250, and 1000 µg/mL) and the negative control group. However, CP significantly (*p*  <  0.001) decreased the MMP%, compared with the negative control (Figure 5B). This reduction in MMP% indicates the possibility of CP triggering the intrinsic pathway of apoptosis, which corroborates the pro-apoptotic effect of CP recorded in this study. Interestingly, PHF prevented the detrimental effect of CP by significantly (*p*  <  0.01) increasing the MMP%, with the dose 250 µg/mL being the most effective. Additionally, the involvement of TRPV1 and TRPM2 channels was investigated by co-treating the cells with PHF and CAP, CPZ, CHPx, or ACA. In all groups incubated with CP, CAP, and/or CHPx, the MMP% was significantly (*p*  <  0.001) lower compared to the negative control group. Similarly, CAP promotes mitochondrial dysfunction by mobilizing extracellular calcium accumulation via TRPV1 channels [35]. In the cells co-treated with PHF and CAP, or PHF and CHPx, we found that the inhibitory effect of PHF on MMP was more pronounced in the PHF + CAP group, indicating the inhibitory activity of PHF on TRPV1 channels (Figure 5B and Figure S2). This result is consistent with our previous report on the MMP inhibitory effect of *Guibourtia tessmannii* via TRPV1 inhibition [19].

#### 2.3.5. ROS Generation Through TRPV1 and TRPM2 Activation in TM3 Cells

The TM3 cells were incubated for 24 h with PHF (62.5, 250, and 1000 µg/mL) in the absence or presence of CP (1600 µg/mL) and/or TRPV1 and TRPM2 agonists and antagonists, and the % of ROS generation was estimated by DCF-DA. According to the results obtained, the % of ROS generation was low in the control and PHF-treated groups. In contrast, CP significantly (*p*  <  0.001) increased the % of ROS generation, compared to the negative control group (Figure 5C and Figure S3). However, the % of ROS generation was significantly (*p*  <  0.05) decreased in the cells co-treated with CP and PHF, in comparison to the CP group. We further co-treated the cells with CAP (a TRPV1 agonist), CPZ (a TRPV1 antagonist), CHPx (a TRPM2 agonist), or ACA (a TRPM2 antagonist) to determine the involvement of TRPV1 and TRPM2 channels. CAP and CHPx activated ROS generation, while CPZ and ACA decreased it. The % of ROS generation was high in the presence of CP, but PHF prevented this action. Since PHF strongly inhibited the activity of CAP and moderately that of CHPx, the ROS scavenging activity of PHF could be mainly mediated through TRPV1 inhibition.

#### 2.3.6. TM3 Cell Migration

**Figure 6:**
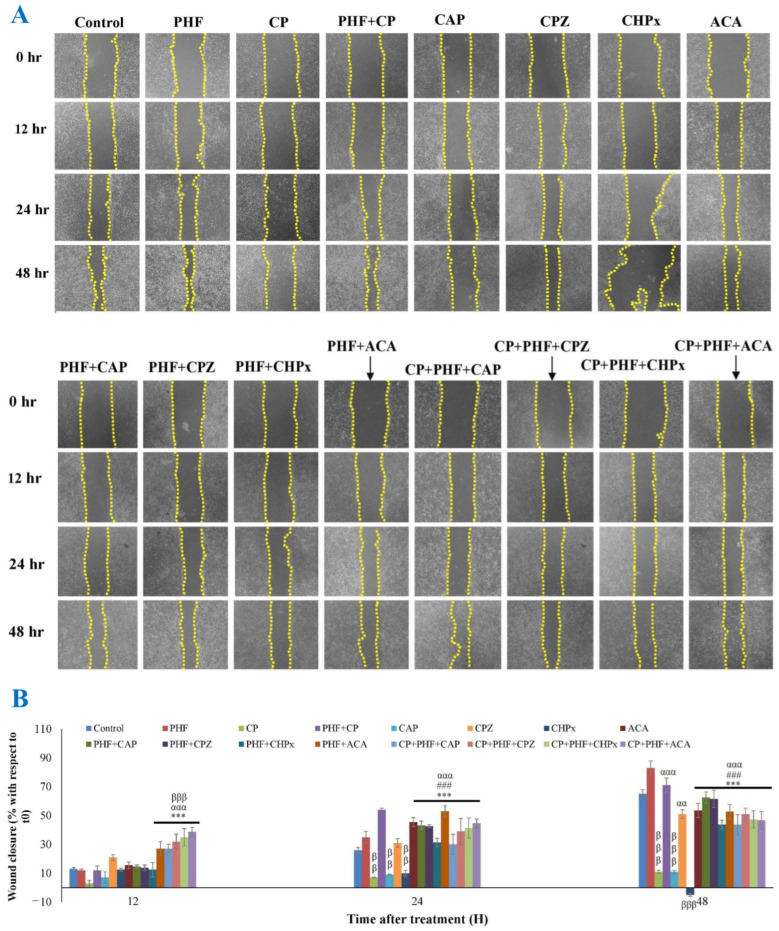
TM3 cell migration assay. (**A**) TM3 cell images were captured at 0, 12, 24, and 48 h. (**B**) The percentage of the scratch area in TM3 cells. For each treatment, the percentage at 12, 24, and 48 h was estimated relative to the percentage of the scratch area of each treatment at T0, considered as 100%. Each bar represents the mean ± SEM. In the control group, the TM3 cells were cultured in normal growth medium. PHF improved the migration of TM3 cells in a dose and time-dependent manner. The co-administration of PHF and CP, PHF and CAP, or PHF and CHPx increased the migration of TM3 cells, compared to CP, CAP, or CHPx groups, respectively. PHF: polyherbal formulation (250 µg/mL); CP: cyclophosphamide (250 µg/mL); CAP: capsaicin (0.01 mM); CPZ: Capsazepine: (0.1 mM); CHPx: cumene hydroperoxide (1 mM); ACA: N-(p-amylcinnamoyl)anthranilic acid (25 μM). ^ββ^ *p* < 0.01; ^βββ^ *p* < 0.001: compared to control; ^αα^ *p* < 0.01; ^ααα^ *p* < 0.001: compared to CP; ^###^ *p* < 0.001: compared to CAP; *** *p* < 0.001: compared to CHPx.

CP is an anticancer drug widely used in modern medicine, but it may negatively affect the migration of healthy cells [36]. Therefore, in this work, the effect of PHF and/or CP on cell migration was determined using a scratch assay. Our results revealed that PHF enhanced TM3 cell migration in a time-dependent manner (Figure 6A,B). After 48 h of treatment with PHF, the wound closure ratio was 83%, while 65% of scratch closure was recorded in the control group. In contrast, we found that CP induced a decline in cell migration, which is consistent with the work of Awadallah et al. [36] on olfactory cells. In the cells co-treated with CP and PHF, the cell migration was significantly (*p*  <  0.01) improved, compared with the CP group. Indeed, the scratch closure ratio after 12, 24, and 48 h of treatment with CP was 3%, 7%, and 11% vs. 13%, 54%, and 71% in the CP + PHF group. On the other hand, CAP and CHPx decreased the cell migration relative to the control. In parallel, it has been demonstrated that CAP decreases the migration of T24 cells by inhibition of SIRT1 [37]. The scratch closure % in TM3 cells after exposure to CAP (0.01 Mm) for 12, 24, and 48 h was 7%, 9%, and 11%, respectively, while Islam et al. [37] showed a migration rate of 58%, 20% and 11% in T24 cells after 24 h of incubation with CAP at doses 10, 100 and 200 µM, respectively. On the other hand, cell migration was improved in the cells co-treated with PHF and CHPx, compared to the CHPx group. After 48 h of treatment, a significant (*p*  <  0.001) increase in TM3 cell migration was recorded in CAP + PHF and CHPx + PHF groups in the presence or absence of CP, compared with CAP or CHPx alone, respectively. CPZ and ACA showed only moderate activities in promoting TM3 cell migration, compared to PHF. However, the combination of PHF and CPZ or PHF and ACA significantly (*p* < 0.001) increased the migration of TM3 cells in comparison to CP, CAP, or CHPx groups. Overall, these data demonstrated the ability of PHF to enhance TM3 cell migration in the presence or absence of CP (Figure 6A,B). It is plausible that PHF may stimulate the migration of TM3 cells by inhibiting TRPV1 and TRPM2 channels. PHF could be exploited as an adjuvant therapy to counteract the detrimental effects of CP on healthy Leydig cells during chemotherapy, but more studies are needed.

### 2.4. Cytotoxicity Study of PHF

**Figure 7:**
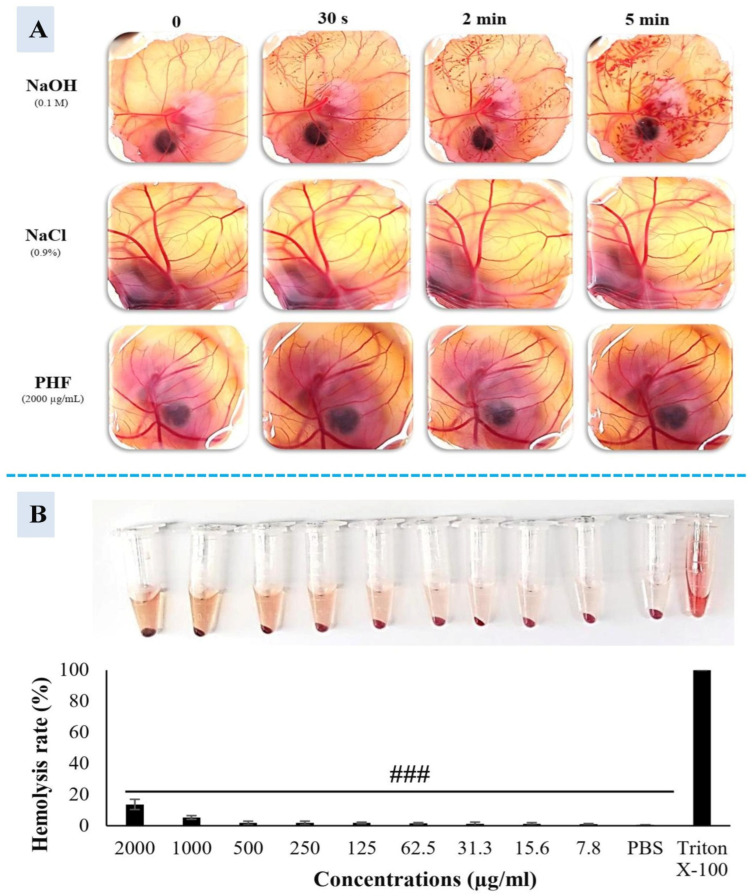
Cytotoxicity of PHF. (**A**). HET-CAM assay. NaOH (0.1 M) strongly irritated the chorioallantoic membrane (presence of hemorrhage, vascular damage, and coagulation), while 0.9% NaCl and PHF did not cause any sign of toxicity 5 min post-application. (**B**). Hemolysis assay. Triton X was hemolytic while PBS and PHF (7.8–500 µg/mL) were non-hemolytic. Experiments were performed in triplicate. Each bar represents the mean ± SEM. ^###^ *p* < 0.001: compared to Triton X-100.

The cytotoxicity study of PHF was conducted by evaluating HET-CAM irritation, TM3 cell morphological changes, and hemolysis. The result of the HET-CAM assay is shown in Figure 7A and Table 3. Indeed, NaOH (0.1 M) strongly irritated the chorioallantoic membrane after application. This was associated with the presence of hemorrhage and vascular damage after 30 s and 2 min of treatment. The irritation was more severe 5 min post-application (presence of vascular damage, hemorrhage, and coagulation). Interestingly, 0.9% NaCl and PHF did not cause any signs of toxicity after 5 min of treatment. In this study, we have also tested the effect of CP and PHF on TM3 cell morphology. In the control group, cells were tightly packed (tp), normally shaped (ns) (polygonal), well organized (wo) with a similar size (ss), and organized plasma membrane (opm) (Figure S4i–iv), as reported earlier [38,39]. In contrast, the cell population in the CP-treated group was lower compared to the control or PHF groups. Moreover, the cells presented an abnormal shape (as) with irregular contour (ic) and membrane blebbing (mb). A lot of floating cells (fc) and cellular debris (cd) were also observed. After 48 h of incubation with CP, the majority of cells had irregular shapes, lost cell–cell contacts, and became atrophic with fuzzy edges. Additionally, the plasma membrane was damaged, and the content of the cell was expelled. As shown in Figure S4iv–xii, PHF did not cause any significant morphological changes in TM3 cells after 24 and 48 h of treatment. Indeed, as observed in the control group, the cells were polygonal in shape, with clear boundaries and complete morphology (Figure 6Biv–xii). Hemolysis assay is a reliable experiment that determines the cytotoxicity of drugs on red blood cells (RBCs) [40]. In this study, the RBCs were exposed to various concentrations of PHF (7.8–2000 µg/mL), Triton X-100 (positive control), or PBS (0.1 M) (negative control), and their hemolytic activity was determined. We found that PHF was non-hemolytic at doses of 7.8 to 1000 µg/mL and slightly hemolytic at doses of 2000 µg/mL, according to ASTM F756-00 norms [41]. However, at all doses, the hemolytic effect of PHF was significantly (*p* < 0.001) lower than that of Triton X-100. Thus, PHF at low or moderate concentrations has no toxic effects on the RBC membrane (Figure 7B).

### 2.5. Fictive Ejaculation Study in Spinal Cord Transected Rats

**Figure 8:**
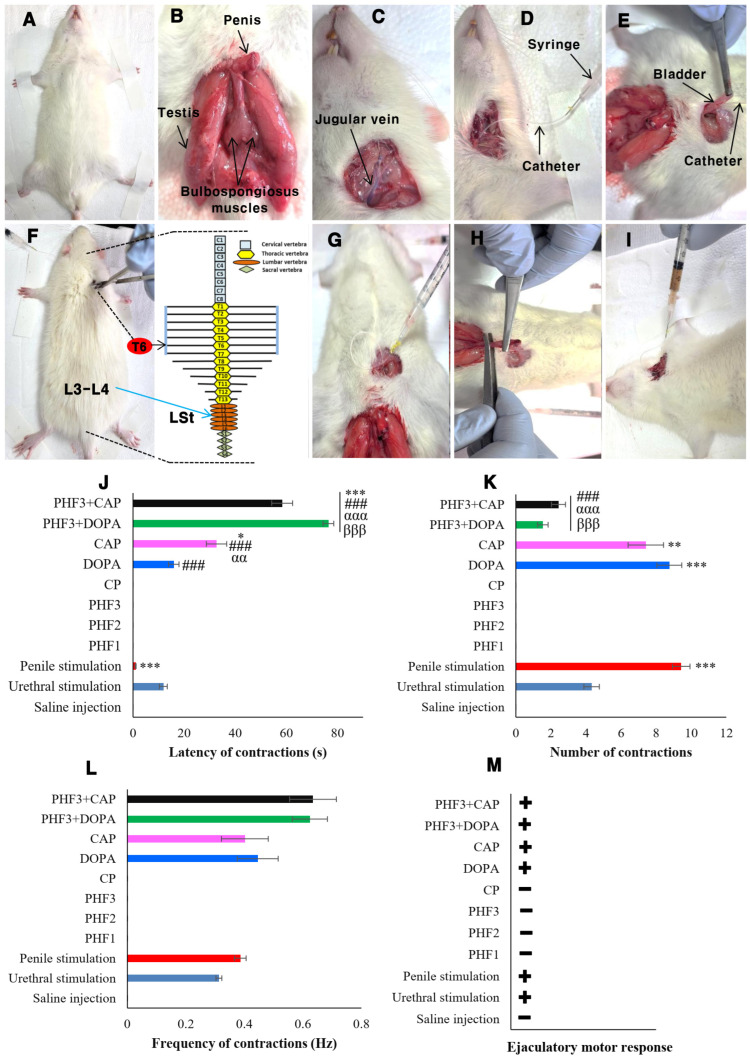
Effects of various treatments on fictive ejaculation in spinal rats. Animals were anesthetized using urethane (1.5 g/kg) (**A**), and the bulbospongiosus muscles were identified and exposed (**B**). The jugular vein was also identified (**C**) and catheterized (**D**), while another catheter was introduced into the pelvic urethra (**E**) for the intravenous injection of drugs and urethral stimulation, respectively. The spinal cord was transected around the T6 segment (**F**), and the animal was prepared for EMG recording as described in the Supplementary Materials (S1). In all rats, urethral (**G**) and penile stimulations (**H**) were performed before the intravenous injection of drugs (**I**). (**J**–**L**). Latency, number and frequency of contractions of the bulbospongiosus muscles, respectively. (**M**). Ejaculatory motor response after mechanical (urethral and penile stimulations) and pharmacological (injection of drugs) stimulations. Number of rats per group (4). All values are expressed as mean ± SEM. * *p* < 0.05, ** *p* < 0.01, *** *p* < 0.001: compared with urethral stimulation; ^###^ *p* < 0.001: compared with penile stimulation; ^αα^ *p* < 0.01, ^ααα^ *p* < 0.001: compared with dopamine; ^βββ^ *p*: < 0.001 compared with capsaicin. LSt: lumbar spinothalamic; saline solution (1 mL/kg); dopamine (10 μg/kg); CP: cyclophosphamide (1 mg/kg); CAP: capsaicin (10 μg/kg); PHF1, PHF2, PHF3: polyherbal formulation at the doses 2.5, 5, and 10 mg/kg, respectively.

In the in vitro section of this work, we found that PHF prevented CP cytotoxicity in TM3 Leydig cells, probably through TRPV1/TRPM2 inhibition. CP chemotherapy is commonly associated with numerous adverse effects, including ejaculatory dysfunctions. Leydig cells play a major role in ejaculatory function because they are the main source of testosterone production, which promotes arousal and regulates ejaculation. Given the protective role of PHF against CP cytotoxicity in Leydig cells observed in vitro, we subsequently investigated the effects of PHF on the ejaculatory process in spinal cord transected rats. Furthermore, the involvement of dopamine receptors and TRPV1 channels was investigated using dopamine and capsaicin as their respective agonists. Fictive ejaculation is a reliable method to explore the effects of mechanical (urethral and penile stimulations) and pharmacological (injection of drugs) stimulations on the spinal generator of ejaculation [13]. Animals were anesthetized with urethane (1.5 g/kg, i.p) (Figure 8A). An incision on the perineum was performed, and the bulbospongiosus muscles were identified and exposed (Figure 8B). The jugular vein was also identified (Figure 8C) and catheterized (Figure 8D), while another catheter (PE-50) was introduced into the pelvic urethra (Figure 8E) for the intravenous injection of drugs and urethral stimulation, respectively. The spinal cord was transected around the T6 segment (Figure 8F), and the animals were prepared for electromyographic (EMG) recording as described in the Supplementary Materials (S1). The spinal cord was transected around the T6 segment to suppress the influence of the supraspinal areas on the ejaculation generator located at L3-L4. Under normal physiological conditions, the supraspinal areas secrete serotonin, which has a predominantly inhibitory tone on the spinal generator of ejaculation [42]. In all rats, urethral (Figure 8G) and penile (Figure 8H) stimulations were performed before the intravenous injection of drugs (Figure 8I).

#### 2.5.1. Effects of Urethral and Penile Stimulations, and the Intravenous Injection of Saline Solution, CP, PHF, Dopamine, and Capsaicin on the Generator of Ejaculation in Spinal Rats

Urethral and penile stimulations induced fictive ejaculation in spinal cord transected rats, characterized by the rhythmic contractions of the bulbospongiosus muscles with an average of 4.32 ± 0.44 and 9.43 ± 0.5 contractions, respectively (Figure 8G,H,L). These contractions (EMG), recorded ex copula, represent the expression of the expulsive phase of ejaculation. Penile stimulation was more effective than urethral stimulation, as reported earlier [13]. Indeed, urethral stimulation was done by injecting a saline solution into the pelvic urethra to increase intraurethral pressure and cause urethral distension, which occurs during the emptying of the contents of the accessory glands into the posterior urethra. The penile stimulation aimed to stimulate the afferent pelvic somatic fibers (pudendal nerve) to generate effective stimulation intended to activate the ejaculatory center located between L3 and L4, and essentially made up of LSt cells [42]. NaCl, 0.9%, used as a negative control, had no effect after injection. On the other hand, the intravenous administration of CP (1 mg/kg) or PHF (2.5, 5, and 10 mg/kg) did not contract the bulbospongiosus muscles 5 min after application. The absence of contractions is not necessarily assimilated to an absence of activity on the ejaculatory center, since PHF may have an inhibitory activity on LSt cells. On the contrary, dopamine and capsaicin, used as positive controls, exhibited pro-ejaculatory activity after injection. Dopamine induced fictive ejaculation more quickly (latency: 15.95 ± 2.04 s) and more powerfully (8.75 ± 0.71 contractions) than capsaicin (latency: 32.67 ± 3.98 s; 7.41 ± 1.01 contractions) (Figure 8J–M). Indeed, both at the central and peripheral levels, dopamine facilitates the ejaculatory response, and when administered intravenously, it stimulates the contraction of the bulbospongiosus muscles [43]. Capsaicin is the main compound of chili pepper. The use of capsaicin for the treatment of retarded ejaculation has been suggested by Pelayo et al. [44], due to its ability to reduce the ejaculation latency in copula. Thus, capsaicin is able to facilitate ejaculation in copula and ex copula.

#### 2.5.2. Effects of PHF on the Pro-Ejaculatory Activity of Dopamine and Capsaicin in Spinal Rats

Since PHF did not activate fictive ejaculation in spinal rats, its effect on the pro-ejaculatory activity of dopamine and capsaicin (a TRPV1 agonist) was studied to explore the possible involvement of dopamine and TRPV1 receptors. Indeed, rats were intravenously co-administered with PHF and dopamine or capsaicin at 3 min intervals, and fictive ejaculation was determined. LSt cells are known as the spinal generator center of ejaculation, and they contain a lot of dopamine and TRPV1 receptors, which are essential for the ejaculatory reflexes and pain [43,45]. We found that PHF significantly (*p* < 0.001) inhibited the pro-ejaculatory action of dopamine and capsaicin. Indeed, the number of discharges of the bulbospongiosus muscles in the PHF + DOPA (1.54 ± 0.3) and PHF + CAP (2.43 ± 0.74) groups were significantly (*p* < 0.001) lowered, by 17.6% and 32.79%, compared with the DOPA (8.75 ± 0.71) and CAP (7.41 ± 1.01) groups, respectively. Furthermore, PHF delayed the pro-ejaculatory action of capsaicin (by 32.69%) better than that of dopamine (17.6%), but no significant changes (*p* > 0.05) in the frequency of contractions were recorded. This inhibitory activity of PHF is comparable to that of *M. whitei* [12], *A. floribunda* [46], and *B. engleriana* [47] recorded in spinal cord transected rats. Since PHF inhibited the pro-ejaculatory activity of dopamine and capsaicin, PHF could act through dopamine and TRPV1 receptors. However, other pathways (like the serotonergic pathway) could be explored because the preventive effect of PHF was not total. Thus, PHF could be a potential candidate for the treatment of premature ejaculation.

### 2.6. Pharmacokinetics and ADME Properties of Compounds Found in PHF

To evaluate the possibility of PHF being a drug candidate, the pharmacokinetics and ADME properties of all compounds detected in PHF were determined using the SwissADME method. According to the results, the compounds did not violate Lipinski’s rules, and their total polar surface area (TPSA) was below 131.6 Å2, indicating their possibility to be exploited as an oral drug, as previously reported [48,49]. Twenty-one compounds possessed high GI absorption capability. A potential drug candidate for oral administration should have a high GI absorption capability to facilitate its activity at the cellular level. Assessment of blood–brain barrier (BBB) permeability is important to predict the safety of a compound on the nervous system [50]. As shown in Table S3, 12 compounds can penetrate the BBB, which may have a side effect on the brain, while 16 compounds cannot cross the BBB. The number of hydrogen bond donors and acceptors of a compound can predict its absorption and permeation potential [48]. Thus, the high permeation potential of some compounds, such as Pyrocatechol, erythritol, quinic acid, Alpha-methyl-DL-phenylalanine, N-(2-thienylmethyl)-2-pyridinamine, hydroquinone, and norephedrine, could be due to the high number of hydrogen bond donors. Additionally, we noticed that Log P values of all components were within the normal range (≤5), except for (Tetrahydroxy cyclopentadienone) tricarbonyliron (0) (Log P = 5.61). Indeed, compounds with Log P ≤ 5 usually have good solubility and absorption, which can facilitate their pharmacological activity. The bioavailability scores of all compounds were within the normal range (0.55–0.85) (Table S3). Components of pharmacological importance exhibiting high GI absorption, high relative abundance (>0.1%), and satisfying all principles of Lipinski drug-likeness properties were selected for molecular docking.

### 2.7. Molecular Docking Between Selected Compounds from PHF Against TRPV1 (PDB ID: 5IS0)

**Figure 9:**
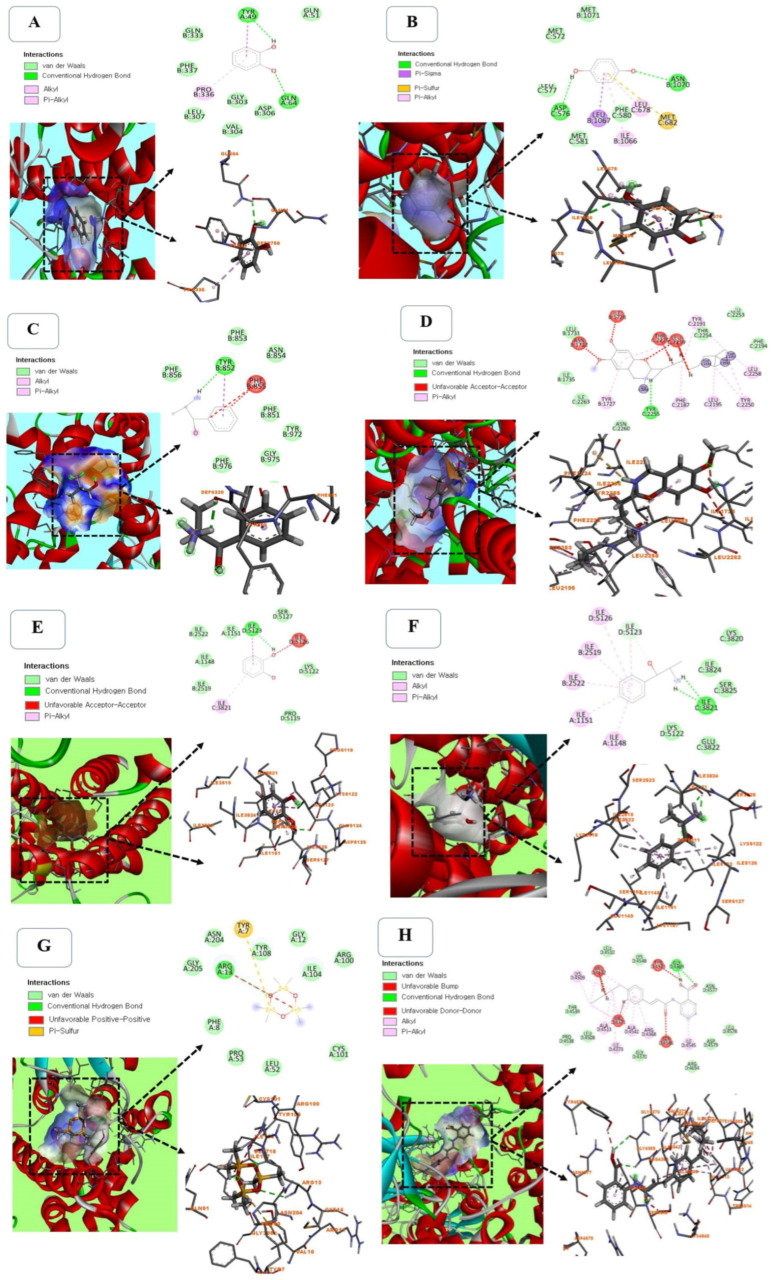
Three-dimensional and two-dimensional diagrams of molecular docking between selected compounds from PHF and TRPV1 (**A**–**D**) and TRPM2 (**E**–**H**). (**A**): Molecular interaction between Pyrocatechol and TRPV1 showing a strong interaction via 2 conventional hydrogen bonds with Tyr-49 and Gln-64, and various van der Waals interactions. (**B**): Docking results of hydroquinone interacting with TRPV1 via 2 conventional hydrogen bonds with Asp-576 and Asn-1070, one Pi-Sulfur interaction with Met-682 residue, and various van der Waals interactions. (**C**): Molecular interaction between (-)-norephedrine and TRPV1 showing one conventional hydrogen bond with Tyr-852 residue and various van der Waals interactions. (**D**): Molecular interaction between Capsazepine and TRPV1 showing one conventional hydrogen bond with Tyr-2255 residue, and various Pi-Alkyl and van der Waals interactions. (**E**): Molecular interaction between Pyrocatechol and TRPM2 showing one conventional hydrogen bond with amino acid Ile-5123, one Pi-Alkyl interaction with ILE-3821 residue, and various van der Waals interactions. (**F**): Binding poses of (-)-norephedrine interacting with TRPM2 through two conventional hydrogen bonds with Ile-3821 residue, one carbon–hydrogen bond with Ile-5123 residue, and various Pi-Alkyl and van der Waals interactions. (**G**): Docking results of Alpha-methyl-DL-phenylalanine interacting with TRPM2, showing one conventional hydrogen bond with Arg 13 residue and a strong van der Waals interaction. (**H**): Molecular interaction between ACA and TRPM2 showing two conventional hydrogen bonds with Gly-4369 and various van der Waals and Alkyl/Pi-Alkyl interactions.

Molecular docking is a reliable and cost-effective approach to studying the molecular interaction of drugs (ligands) with their targets (proteins). Since the TRPV1 channel is involved in various physiological processes [51,52], the selected compounds from PHF were docked against TRPV1 (PDB ID: 5IS0) to evaluate their binding affinity and molecular interaction. Capsazepine (a TRPV1 inhibitor) was used as a reference ligand. We found that the binding poses of ligands with the active pocket of TRPV1 depend on the ligand structure (Figure 9A–F). For example, Pyrocatechol displayed a strong interaction (−7.73 kcal/mol) with the active site of TRPV1 via two conventional hydrogen bonds with Tyr-49 and Gln-64, one hydrophilic interaction with Pro-336, and various van der Waals interactions with Gln-333, Phe-337, Leu-307, Val-304, Gly-303, Asp-306, and Gln-51 residues (Figure 8A, Table S4). p-Menthone (−9.83 kcal/mol), Eucalyptol (−8.69 kcal/mol), and Pulegone (−10.06 kcal/mol) displayed a strong binding affinity with the proteins without a conventional hydrogen bond, but it was slightly lower than that of Capsazepine (−11.25 kcal/mol), used as a selective TRPV1 agonist. Hydroquinone interacted with TRPV1 via two conventional hydrogen bonds with Asp-576 and Asn-1070 residues, one Pi-Sulfur interaction with Met-682 residue, and various van der Waals interactions, with a binding energy of −7.99 kcal/mol (Figure 9B, Table S4). As shown in Figure 9C, (-)-norephedrine was attached to the binding site of TRPV1 via one conventional hydrogen bond with Tyr-852 residue. However, Capsazepine, used as a reference inhibitor, exhibited the highest affinity (−11.25 kcal/mol) and interacted with TRPV1 via one conventional hydrogen bond with Tyr-2255 residue, and various Pi-Alkyl and van der Waals interactions (Figure 9F). Hydroquinone has good stability, due to the presence of two conventional hydrogen bonds. Hydroquinone has various pharmacological benefits, including antimicrobial [53], anti-inflammatory [54], skin lightening [55], anticancer [56], pro-apoptotic [57], and antioxidant [58] activities (Table S4). The presence of this compound in PHF could justify its ability to prevent the detrimental effects of CP in TM3 Leydig cells observed in this study. Among all the selected compounds detected in PHF, N-(2-thienylmethyl)-2-pyridinamine showed the best binding affinity (−10.02 kcal/mol) with the active site of TRPV1, but it was slightly lower compared with Capsazepine, a TRPV1 blocker (−11.25 kcal/mol) (Table S4).

### 2.8. Molecular Docking Between Selected Compounds from PHF Against TRPM2 (PDB ID: 6PUS)

To predict the mechanism of PHF in preventing CP cytotoxicity, molecular docking of major compounds from PHF against TRPM2 was performed. Indeed, TRPM2 activation induces oxidative stress by increasing cytoplasmic Ca^2+^ accumulation and cellular damage, which may lead to cell death [59,60]. In the present study, there was no conventional hydrogen bond between p-Menthone, Eucalyptol, or Pulegone and the active pocket of TRPM2, but they exhibited a high binding affinity and various Alkyl/Pi-Alkyl and van der Waals interactions. Pyrocatechol created one conventional hydrogen bond with amino acid Ile-5123, one Pi-Alkyl interaction with ILE-3821 residue, and various van der Waals interactions, while (-)-norephedrine (−8.60 kcal/mol) interacted with the active pocket of TRPM2 through two conventional hydrogen bonds (Ile-3821), which is assumed to enhance its binding affinity (Figure 9E,F and Table S4). Alpha-methyl-DL-phenylalanine interacted with TRPM2 via one conventional hydrogen bond with Arg-13 residue and several van der Waals interactions (Figure 9G and Table S4). ACA, used as a TRPM2 inhibitor, interacted with the active pocket of TRPM2 via two conventional hydrogen bonds with Gly-4369 residue, and various van der Waals and Alkyl/Pi-Alkyl interactions, and exhibited the best binding affinity (−14.52 kcal/mol) (Figure 9H and Table S4). Since p-Menthone, Eucalyptol, and (-)-norephedrine displayed strong stability with the protein, they are potential TRPM2 inhibitors, which may further support the activities of PHF. Overall, the binding affinity of selected compounds from PHF against TRPV1 was higher than that of TRPM2. These results corroborate the in vitro findings. The strong binding interaction of selected compounds from PHF against TRPV1 demonstrated in silico shows that PHF could exhibit its antioxidant, anti-lipid peroxidation, and antiapoptotic activity through TRPV1 channels. However, more mechanistic studies are required to confirm this action.

**Figure 10:**
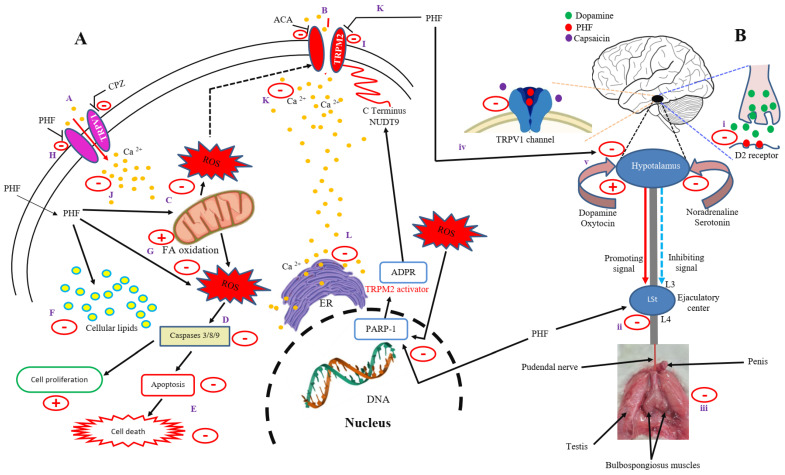
Possible mechanism of action of PHF. ACA and CPZ inhibited TRPM2 and TRPV1 channels, respectively, while PHF inhibited both channels, with the greatest effect observed on TRPV1 (**A**,**B**). PHF could diffuse directly into the cell, scavenge cytosolic ROS and/or prevent ROS overproduction by mitochondria, which could prevent the production of pro-apoptotic factors, leading to the inhibition of apoptosis and cell death (C–E). Additionally, PHF could exert its anti-lipid peroxidation activity by decreasing cellular lipids and increasing mitochondrial fatty acid oxidation, which in turn could exacerbate its antioxidant potential (F–G). On the other hand, PHF may act as a TRPV1 and TRPM2 blocker (H–I). By inhibiting these channels, PHF could prevent extracellular calcium influx, as they are calcium-permeable channels (J–K). This could decrease the intracellular calcium level and inhibit endoplasmic reticulum calcium release and mitochondrial activity, which in turn could inhibit ROS production, apoptosis, and cell death (L, D, E). On the other hand, we found that PHF prevented the pro-ejaculatory activities of dopamine and capsaicin. It can be suggested that PHF could bind to D2 receptors at the hypothalamus and spinal cord and prevent its activation by its natural agonist (dopamine), leading to inhibition of the ejaculatory center, which can justify the significant reduction in the number of contractions of the ejaculatory muscles observed in this study (i–iii). Moreover, TRPV1 channels, densely expressed in the spinal cord, are mainly involved in pain regulation, but studies show that the activation of TRPV1 by capsaicin reduces the ejaculatory latency. PHF may also act through TRPV1 inhibition, since we found that it inhibited the pro-ejaculatory action of capsaicin (iv). Additionally, the inhibition of TRPV1 by PHF could prevent dopamine release and delay the ejaculatory reflex (v).

The possible mechanism of action of PHF is summarized in Figure 10A,B. Activation of TRPV1 and TRPM2 channels is known to increase cytosolic calcium accumulation and promote mitochondrial membrane potential and ROS overproduction, leading to oxidative stress, apoptosis, and cell death. DNA damage can also cause oxidative stress through PARP-1, ADPR, and TRPM2 (NUDT9 region in the C domain) activation [61]. However, ACA and CPZ inhibited TRPM2 and TRPV1 channels, respectively, while PHF inhibited both channels, with the greatest effect observed on TRPV1 (A, B). Based on the results obtained, PHF could diffuse directly into the cells, scavenge cytosolic ROS, and/or prevent ROS overproduction by mitochondria, which could prevent the production of pro-apoptotic factors, leading to the inhibition of apoptosis and cell death (C–E). Additionally, PHF could exert its anti-lipid peroxidation activity by decreasing cellular lipids and increasing mitochondrial fatty acid oxidation, which in turn could exacerbate its antioxidant potential (F–G). On the other hand, PHF may act as a TRPV1 and TRPM2 blocker (H–I). Indeed, in the present study, the activities of CHPx (TRPM2 agonist) and CAP (TRPV1 agonist) were inhibited by PHF, indicating the involvement of these channels. By inhibiting these channels, PHF could prevent extracellular calcium influx, as they are calcium-permeable channels (J–K). This could decrease the intracellular calcium level and inhibit endoplasmic reticulum calcium release and mitochondrial activity, which in turn could inhibit ROS production, apoptosis, and cell death (L, D, E) (Figure 10A). On the other hand, we found that PHF prevented the pro-ejaculatory activities of dopamine and capsaicin. The spinal generator of ejaculation is controlled by the supraspinal sites through various neurotransmitters, which can inhibit (via noradrenaline and serotonin) or activate (via dopamine and oxytocin) the ejaculatory center through a complex mechanism. Dopamine promotes ejaculation by activating D2 receptors, while the activation of the serotonergic pathway could delay ejaculation when the presynaptic 5-HT2c receptors are stimulated or shorten ejaculatory latency when the postsynaptic 5-HT1a receptors are activated [62]. Based on the results obtained, it can be suggested that PHF could bind to D2 receptors at the hypothalamus and spinal cord and prevent its activation by its natural agonist (dopamine), leading to inhibition of the ejaculatory center, which can justify the significant reduction in the number of contractions of the ejaculatory muscles observed in this study (Figure 10B i–iii). Moreover, TRPV1 channels, densely expressed in the spinal cord, are mainly involved in pain regulation, but studies show that the activation of TRPV1 by capsaicin reduces ejaculatory latency [44]. PHF may also act through TRPV1 inhibition, since we found that it inhibited the pro-ejaculatory action of capsaicin (iv). Additionally, activation of TRPV1 channels is known to increase dopamine release from dopamine neurons [63]. Thus, the inhibition of TRPV1 by PHF could prevent dopamine release and delay the ejaculatory reflex (v). However, PHF may also delay ejaculation through the serotonergic pathway, but this action was not evaluated in this study.

## 3. Materials and Methods

### 3.1. Preparation of PHF, GC-MS Analysis, and Quantification of Total Phenolics (TP) and Total Flavonoids (TF)

The botanical information of all plants is summarized in Table 4. As recommended by the traditional healers, the plants were shade-dried individually for 2 weeks, and then ground into powder. A total of 1 kg (200 g of each plant) of powder was macerated in 5 L of distilled water for 48 h, filtered using Whatman No. 1 filter paper, and the filtrate was evaporated at 55 °C for 72 h. Ninety-eight grams of extract (PHF) were obtained after evaporation (extraction yield 9.80%). The GC-MS analysis and quantification of TP and total TF in PHF are described in the Supplementary Materials (S1). 

### 3.2. In Vitro Studies

#### 3.2.1. Antioxidant Study and Measurement of Lipid Peroxidation Inhibition

The experimental protocol for the determination of DPPH and ABTS^+^ free radical scavenging activity, as well as CUPRAC and FRAP reducing power, and the level of lipid peroxidation inhibition of PHF are described in the Supplementary Materials (S1).

#### 3.2.2. Cell Culture, Cell Viability, and Treatments

TM3 cells were obtained from the Korean Cell Line Bank (KCLB, Seoul, ROK) and cultured according to the suppliers’ instructions. The passage process was done twice a week at about 80–90% growth confluence. The cells were exposed for 24 h to various concentrations of PHF and/or CP, and the cell viability was estimated by using 3-(4,5-di-methylthiazol-2-yl)-2,5-diphenyltetrazolium bromide (MTT) assay. The cells were then divided into 20 groups (density of 1 × 106 cells/flask) and treated as follows: (1)-control: cells without any treatment; (2–4)-PHF: cells treated with PHF at 1000, 250, and 62.5 mg/mL; (5)-CP: cells incubated with CP at 250 µg/mL; (6–8)-CP + PHF: cells co-treated with CP and PHF at 1000, 250, and 62.5 mg/mL; (9)-CAP: cells treated with CAP (a TRPV1 agonist) at 0.01 mM; (10)-CPZ: cells incubated with CPZ (a TRPV1 antagonist) at 0.1 mM; (11)-CHPx: cells exposed to CHPx (TRPM2 activator) at 1 mM; (12)-ACA: cells treated with ACA (a TRPM2 blocker) at 25 μM; (13–16)-PHF + TRPV1 or TRPM2 activators or blockers: cells co-treated with PHF + CAP, PHF + CPZ, PHF + CHPx, and PHF + ACA; (17–20)-CP + PHF + TRPV1 or TRPM2 activators or blockers: cells pretreated with CP and exposed to PHF + CAP, PHF + CPZ, PHF + CHPx, and PHF + ACA. At the end of the treatment period (24 h), various biological parameters such as oxidative stress-related parameters (DPPH, ABTS, Cupric and Ferric reducing power, and ROS generation), apoptosis, mitochondrial membrane potential, and cell migration were determined. The doses of CP, CAP, CPZ, CHPx, and ACA were selected from previous studies [19,38,64].

#### 3.2.3. Cytotoxicity Assay: Hemolysis, HET-CAM Irritation Ex Vivo, and Cell Morphology

The cytotoxicity study of PHF was determined through a hemolysis assay using human red blood cells, an HET-CAM irritation ex vivo toxicity assay using fertilized eggs, and cellular morphological changes detected microscopically, as described in the Supplementary Materials (S1).

### 3.3. In Vivo Studies

#### 3.3.1. Animals and Experimental Treatments

Adult male Wistar rats (body weight: 300–320 g) were purchased from Central Lab Animal Inc. (Seoul, Republic of Korea) and maintained (four rats per cage) for a week under a natural LD cycle with free access to food and water. The study was approved by the Institutional Animal Care and Use Committee (Reference: KW-241024-1). The fictive ejaculation study was performed following the accepted ethical rules described in the European community guidelines [65]. Rats were randomly distributed into 9 groups (n = 4) and treated as follows: (1)-control: rats receiving saline solution (0.1 mL/100 g); (2–4)-PHF1, PHF2, and PHF3: rats receiving polyherbal formulation at doses of 2.5, 5, and 10 mg/kg, respectively; (5)-CP: animals treated with cyclophosphamide (1 mg/kg); (6)-DOPA: rats given dopamine (10 μg/kg); (7)-CAP: rats administered with capsaicin (10 μg/kg); (8)-PHF + DOPA: animals co-treated with PHF (10 mg/kg) and dopamine (10 μg/kg) at 3 min intervals; (9)-PHF + CAP: rats co-administered with PHF (10 mg/kg) and capsaicin (10 μg/kg) at 3 min intervals. For each administration, the infusion time was 5 s.

#### 3.3.2. Surgical Procedure, and Activation and Recording of the Rhythmic Genital Motor Pattern of Ejaculation

The fictive ejaculation study was performed as described in the Supplementary Materials (S1). The electromyographic (EMG) activity of the bulbospongiosus muscles was recorded, and the latency of contractions, the number of discharges, and the frequency of contractions of the ejaculatory muscles were determined [13].

### 3.4. In Silico Studies

#### 3.4.1. Pharmacokinetics and ADME Properties

The pharmacokinetics and ADME properties of all compounds detected in PHF were determined via SwissADME [66]. Briefly, compounds were converted into SMILES format, and various properties, including molecular weight, number of rotatable bonds, number of hydrogen bonds (donor and acceptor), molar refractivity, total polar surface area (TPSA), lipophilicity (Log P), gastrointestinal (GI) absorption, blood–brain barrier (BBB) permeability, Lipinski violations, and bioavailability score were calculated using the SwissADME package. Components with high GI absorption, high BBB permeation, relative abundance higher than 0.1%, and satisfying all Lipinski’s rules were selected for molecular docking.

#### 3.4.2. Molecular Docking

Selected compounds (ligands) were docked against TRPV1 (PDB ID: 5IS0) and TRPM2 (PDB ID: 6PUS) to predict their binding patterns. The ligands were generated using UCSF Chimera software (version 1.16, San Francisco, CA, USA), while the proteins were downloaded from the protein data bank (PDB; www.rcsb.org) (accessed on 30 October 2024) and prepared as described previously [17]. The ligands were docked with the active sites of TRPV1 and TRPM2 proteins using ArgusLab (version 4.0.1), and the results were analyzed using the BIOVIA Discovery Studio Visualizer 2021 client.

### 3.5. Statistical Analysis

The experiments were repeated three times. Results are presented as mean plus or minus standard error of the mean (SEM). Differences among the means were analyzed using one-way ANOVA, followed by the Tukey HSD post hoc test using STATISTICA software (version 8.0, StatSoft, Inc., Tulsa, OK, USA). The significance threshold was established at 0.05 or lower.

## 4. Conclusions

In conclusion, various phytocompounds were detected in PHF by GC-MS analysis. PHF prevented the cytotoxicity of CP in TM3 Leydig cells by inhibiting apoptosis, lipid peroxidation, ROS accumulation, and mitochondrial membrane potential, and improving cell migration and cell proliferation, probably through TRPV1 and TRPM2 inhibition. This finding was supported by the strong binding affinity of the selected phytocompounds detected in PHF with the active pockets of TRPV1 and TRPM2. However, patch-clamp or calcium imaging for these channels would reinforce this finding. This could be considered a limitation of the present study. The cytotoxicity study of PHF through HET-CAM and hemolysis assays revealed no harmful effect after treatment. In spinal male rats, PHF inhibited the pro-ejaculatory effects of CAP by preventing the rhythmic contractions of the bulbospongiosus muscles, indicating TRPV1 receptor inhibition. Overall, PHF could be a potential candidate for the development of an effective therapy for the management of CP chemotherapy-related disorders, such as premature ejaculation.

## Figures and Tables

**Table 1:** Compounds detected in PHF using the gas chromatography/mass spectrometry (GC–MS) method.

Retention Time (Minute)	Area %	Molecular Weight	Molecular Formula	Name of the Compound
4.041	2.97	88.11	C_4_H_8_O_2_	Oxirane, (methoxymethyl)
5.789	16.33	154.25	C_10_H_18_O	p-Menthone
6.230	2.27	98.1	C_5_H_6_O_2_	2-Hydroxycyclopent-2-en-1-one
6.234	7.22	154.25	C_10_H_18_O	Eucalyptol
7.286	2.62	172.16	C_2_H_8_N_2_O_5_S	Carbamimidic acid
7.394	0.64	103.08	C_2_H_5_N_3_O_2_	Urea, n-methyl-n-nitroso-
8.437	0.37	128.13	C_5_H_8_N_2_O_2_	Cycloglycylalanine
8.754	0.82	126.2	C_7_H_14_N_2_	Cyclopentanone, dimethylhydrazone
8.883	0.45	112.17	C_6_H_12_N_2_	Methanamine, n-(1-methyl-2-pyrrolidinylidene)-
9.035	0.88	129.24	C_8_H_19_N	2-Heptanamine, 5-methyl-
9.544	0.85	114.1	C_4_H_6_N_2_O_2_	Maleamide
9.693	0.97	114.19	C_6_H_14_N_2_	N1,N1-dimethyl-n2-isopropylformamidine
9.812	1.97	98.1	C_5_H_6_O_2_	2(3h)-Furanone, 5-methyl-
9.856	0.37	144.12	C_6_H_8_O_4_	4h-Pyran-4-one, 2,3-dihydro-3,5-dihydroxy-6-methyl-
10.613	3.96	110.11	C_6_H_6_O_2_	Pyrocatechol
11.052	0.82	190.27	C_10_H_10_N_2_S	N-(2-thienylmethyl)-2-pyridinamine
11.663	3.08	110.11	C_6_H_6_O_2_	Hydroquinone
12.375	0.39	374.38	C_12_H_18_N_6_O_6_S	5′-O-[n,n-dimethylsulfamoyl]adenosine
12.981	2.38	151.21	C_9_H_13_NO	(-)-Norephedrine
13.940	4.31	104.1	C_4_H_8_O_3_	Tetrahydro-3,4-furandiol
15.766	1.40	122.16	C_8_H_10_O	4,5,6,6a-Tetrahydro-2(1h)-pentalenone
15.995	11.26	192,17	C_7_H_12_O_6_	Quinic acid
16.148	0.68	343.29	C_14_H_17_NO_9_	Tetraacetyl-d-xylonic nitrile
16.189	1.50	179.22	C_10_H_13_NO_2_	Alpha-methyl-DL-phenylalanine
16.240	1.22	122.12	C_4_H_10_O_4_	Erythritol
16.562	1.09	343.29	C_14_H_17_NO_9_	Tetraacetyl-d-xylonic nitrile
16.600	0.36	102.13	C_5_H_10_O_2_	2H-pyran-3-ol, tetrahydro-
19.736	2.36	256.42	C_16_H_32_O_2_	Palmitic acid
21.371	0.84	308.5	C_20_H_36_O_2_	Eicosadienoic acid
21.684	0.75	342.9	C_20_H_35_ClO_2_	Linoleic acid
21.987	2.97	282.5	C_18_H_34_O_2_	Oleic acid
22.613	0.42	283.957	C_8_H_4_FeO_8_	(Tetrahydroxycyclopentadienone)tricarbonyliron(0)
25.582	12.86	98.1	C_5_H_6_O_2_	5-Methyl-2(5H)-furanone
35.412	8.62	152.23	C_10_H_16_O	Pulegone

**Table 2:** IC50 (µg/mL) values for the DPPH, ABTS^+^ FRAP enhancement activities of PHF and AA.

	PHF (IC50: µg/mL)	AA (IC50: µg/mL)
DPPH	124.45	68.89
ABTS	58.16	47.59
FRAP	524.87	75.52

**Table 3:** HET-CAM irritation score test before (T0) and after treatment (30 s to 5 min) with NaOH (0.1 M) (positive control), 0.9% NaCl (negative control), and PHF (2000 µg/mL).

Treatments	HET-CAM (Average)				Irritation Score	Irritation Category
	0 s	30 s	2 min	5 min		
NaOH (0.1 M), positive control	0	5	8	9	22	Strong irritation
0.9% NaCl, negative control	0	0	0	0	0	Non-irritant
PHF (2000 µg/mL)	0	0	0	0	0	Non-irritant

**Table 4:** Botanical information of PHF.

Botanical Name	Vernacular Name	Common Name	Part Used	Family	Voucher Specimen Number	Percentage Used	Period of Collection	Region of Collection (GPS Coordinates)
*Mondia whitei*	Limte	White’s ginger	roots	Apocynaceae	42920/HNC	20	April 2023	Bafoussam (5° 28′ 39.90″ N 10° 25′ 3.32″ E)
*Dracaena arborea*	Keubgouh	African Dragon Tree	stem barks	Asparagaceae	25361/SFR/Cam	20	April 2023	Dschang (5° 26′ 38.29″ N, 10° 3′ 11.95″ E)
*Bridelia ferruginea*	Kimi	-	stem barks	Euphorbiaceae	42920/HNC	20	April 2023	Bangangté (5° 14′ 60.00″ N, 10° 49′ 59.99″ E)
*Guibourtia tessmannii*	Essingang	Bubinga	stem barks	Fabaceae	1037/SRFCA	20	April 2023	Ngoumou (3° 53′ 45.25″ N, 12° 20′ 47.00″ E)
*Helichrysum odoratissimum*	Mbantchuet	Imphepho	whole plant	Asteraceae	HNC 1640	20	April 2023	Dschang (5° 26′ 38.29″ N, 10° 3′ 11.95″ E)

## Data Availability

The original contributions presented in the study are included in the article/Supplementary Materials, further inquiries can be directed to the corresponding author.

## Supplementary Materials

The following supporting information can be downloaded at https://www.mdpi.com/article/10.3390/ph18060803/s1, Table S1: Some pharmacological activities of selected compounds detected in PHF; Table S2: Total phenolic and flavonoid contents in PHF; Table S3: Pharmacokinetics and ADME properties of compounds identified in PHF; Table S4: Molecular docking values of selected compounds from PHF against TRPV1 (PDB ID: 5IS0) and TRPM2 (PDB ID: 6PUS); Figure S1: Apoptotic cells detected by AO/EB staining; Figure S2: RH123 fluorescent images depicting the effects of various treatments on mitochondrial membrane potential in TM3 cells; Figure S3: DCF-DA images depicting the effects of various treatments on ROS generation in TM3 cells; Figure S4: Effects of treatments on cell morphology.

## Abbreviations

ABTS	(2,2′-azino-bis(3-ethylbenzothiazoline-6-sulfonic acid))
ACA	N-(p-amylcinnamoyl)anthranilic acid
ADME	Absorption, Distribution, Metabolism, and Excretion
AO/EB	Acridine Orange/Ethidium Bromide
Bax	Bcl-2-like protein 4
BBB	blood–brain barrier
Bcl-2	B-cell lymphoma 2
CAP	capsaicin
CHPx	cumene hydroperoxide
CP	cyclophosphamide
CPZ	Capsazepine
CUPRAC	Cupric reducing antioxidant capacity
DCF-DA	Diacetyldichlorofluorescein
DPPH	2,2-diphenyl-1-picrylhydrazyl
FRAP	Ferric reducing antioxidant power
GC-MS	gas chromatography/mass spectrometry
GI	gastrointestinal
H_2_O_2_	hydrogen peroxide
HET-CAM	Hen’s Egg Test–Chorioallantoic Membrane
MMP	mitochondrial membrane potential
MTT	3-(4,5-di-methylthiazol-2-yl)-2,5-diphenyltetrazolium bromide
Na_2_CO_3_	sodium carbonate
NaCl	sodium chloride
NaOH	sodium hydroxide
NFκB	Nuclear Factor Kappa B
PBS	phosphate-buffered saline
PDB	protein data bank
PHF	polyherbal formulation
RBCs	red blood cells
ROS	reactive oxygen species
TF	total flavonoids
TNFα	Tumor Necrosis Factor-Alpha
TP	total phenolics
TPSA	total polar surface area
TRPM2	transient receptor potential melastatin 2
TRPV1	transient receptor potential vanilloid 1
