# Supplementary material for: A Novel Polyherbal Formulation Modulates Cyclophosphamide-Induced Cytotoxicity in TM3 Leydig Cells and Delays Fictive Ejaculation in Spinal Cord Transected Male Rats
Source: Pharmaceuticals (Basel). 2025 May 27;18(6):803. doi: 10.3390/ph18060803 (PMC12196003; doi:10.3390/ph18060803)
Supplement: Supplementary file 1 [file pharmaceuticals-18-00803-s001.zip › pharmaceuticals-3650367-supplementary.pdf]

Supplementary material (S1)

Materials and Methods

Gas chromatography/mass spectrophotometry (GC-MS) analysis of PHF

The volatile compounds present in PHF were identified by using a GC (Agilent 789A, Agilent, Santa Clara, CA, USA)/MS (Agilent 5975C; GC-MSD) system, according to the procedure described earlier (Saravanakumar et al., 2021). Briefly, the powdered extracts were diluted in methanol at the concentration of 1 mg/mL. Then,1 μL of the sample was injected in a split mode (scan range of m/z 50–500) in the HP-5 capillary column at 250 °C and helium (99.99 %) gas was used as the carrier at a flow-rate of 1 mL/min. The total running time was 73 min. The compounds found in PHF were identified by matching the GC-MS data of samples with the electronic library of W8N05ST.L.

Quantification of total phenolics and total flavonoids

The estimation of total phenolic content in PHF was carried out using Folin Ciocalteu method. Indeed, 100 μL of each extract (at 1 mg/mL) was added to Folin-Ciocalteu's phenol reagent (200 μL) and Na_2CO_3 (500 μL), and mixed well by gentle shaking. The mixture was incubated for 50 min at 25˚C for color development, and the absorbance was recorded at 760 nm. The standard utilized in this study was gallic acid, and the experiment was conducted three times to ensure accuracy. The total phenolic content was estimated as milligram gallic acid equivalents per 100 g of dry weight (GAE/g DW) (Ainsworth and Gillespie, 2007).

The total flavonoid content of samples was estimated as described by Naveen et al. (2023). Briefly, 100 μL of each extract (at 1 mg/mL) was mixed with distilled water (250 μL) and ethanol (150 μL; 95%). Then, $AlCl_3$ (25 μL; 10%) and CH_3COOK (1M; 25 μL) were added and the mixture was incubated for 30 min at room temperature (25˚C). The absorbance was recorded at 415 nm and the total flavonoid content was calculated and expressed as milligram quercetin equivalent per gram of dry weight (mg QE/g DW) (Apak et al., 2008; Valle et al., 2020).

Antioxidant study and measurement of lipid peroxidation inhibition

DPPH and ABTS$^+$ free radical scavenging activity assay

The DPPH radical was estimated as described earlier (Naveen et al., 2023). Nine concentrations (7.8, 15.6, 31.3, 62.5, 125, 250, 500, 1000, and 2000 μg/mL) of PHF and ascorbic acid were used. Briefly, 100 μl of each sample was added to the DPPH working solution (100 μL). After incubation in a dark at room temperature, the absorbance was recorded at 517 nm and the DPPH free radical scavenging percentage (%) was estimated (Naveen et al., 2023).

The ABTS radical scavenging assay of PHF was estimated according to the literature (Blois, 1958). Briefly, 100 μL of samples were mixed with 100 μL ABTS$^+$, incubated in the dark for 10 min and the absorbance was measured at 734 nm. The % of ABTS$^+$ radical was estimated using methanol and ascorbic acid as control solvent and standard, respectively.

Cupric (CUPRAC) and Ferric (FRAC) reducing antioxidant capacity assay

The CUPRAC assay was done as described in the literature (Apak et al., 2008). Briefly, the mixture (100 μL of extracts + 61 μL $CuCl_2$ solution at 10 mM + 61 μL neocuproine at 7.5 mM + and 61 μL NH_4CH_3COO buffer at pH 7; 1.0 mM) was incubated in the dark for 60 min at 37˚C and the absorbance was recorded at 450 nm. The CUPRAC of samples was estimated, using ethanol and ascorbic acid as control solvent and standard, respectively.

The FRAC of samples were determined based on previous studies (Gao et al., 2020; Sathiyaseelan et al., 2021). Briefly, the samples were mixed with $K_3[Fe(CN)_6]$ (500 μL, 30 mM) and PBS (175 μL, 0.6 M, pH 6.6), incubated for 20 min at 50 °C, added to 500 μL of TCA (10%) and (1500 μL, 0.1%) and the absorbance was recorded at 700 nm. The FRAC of samples was estimated using ascorbic acid as standard (Sathiyaseelan et al., 2021).

Lipid peroxidation inhibition (LPI) assay

The estimation of LPI of samples was carried out using egg yolk homogenate as a lipid-rich media as described previously (Roberto et al., 2000). Briefly, 0.1 ml of samples was added to 0.5 ml of egg yolk homogenate (10%) and the mixture was adjusted to 1 ml with distilled water. $FeSO_4$ (0.07 M, 0.05 ml) was added, and the solution was incubated for 30 min. In addition, 1.5 ml of acetic acid (20%), 1.5 ml of TBA (0.8%) solution prepared SDS (1.1%), and 0.05 ml of TCA (20%) were added. The mixture was heated at 95°C for 60 min and n-butanol (5 ml) was added after cooling. The supernatant was collected after centrifugation

(3000 rpm/10 min) and the absorbance was measured (at 532 nm). The LPI was then estimated (Habu et al., 2015).

Cytotoxicity assay: hemolysis, HET-CAM irritation *ex vivo* and cell morphology

Hemolysis assay

The effects of PHF on hemolysis were tested on red blood cells (RBCs). In brief, the mixture of 1 mL of defibrinated blood and 10 mL of PBS was centrifuged (2000 rpm/10 min) to isolate the RBCs. Subsequently, different concentrations (7.8-2000 μg/mL) of PHF, PBS (used as negative control) and 1% Triton X-100 (used as positive control) were mixed with 200 μL of RBCs suspended in PBS, and the mixture was incubated at 37 °C for one hour. Then, the samples were centrifuged (2000 rpm/10 min), and the absorbance of the supernatant was measured. The hemolytic percentage was calculated as described earlier (Li et al., 2024).

HET-CAM irritation *ex vivo* toxicity assay

This experiment was conducted following the previously described methodology (Jin et al., 2023). Briefly, prior to the experiment, the eggs were examined by candling to ensure the presence of live embryos. Fifteen eggs (3/group) were divided as follows: (1) negative control group treated with 0.9% NaCl; (2) positive control group administered with NaOH (0.1M); and (3-5) plant-treated groups exposed to PHF at concentrations of 500, 1000, 2000 μg/mL, respectively. The shells of the eggs were carefully removed, and the outer membrane was taken off using 30 cm blunt tweezers to expose the chorioallantoic membrane. 200 μL of each sample was applied to the membrane and left for 20 seconds before being wiped off with tissue paper. Toxicity-related parameters such as hemorrhage, vascular damage, and coagulation were observed at 0 s (before treatment), 30 s, 2 min, and 5 min.

Cell morphology

The cells were treated for 24 hours with PHF or CP. After treatment, cells were washed with PBS, fixed with 3.7% formaldehyde, and stained using Alexa Fluor 488 phalloidin and DAPI (Invitrogen, UK). Cell morphology was observed and recorded under an inverted fluorescence microscope (EVOS FL digital inverted fluorescence microscope (AMG)).

***In vivo* study**

Surgical preparation

Animals were urethane-anesthetized (1.5 g/kg intraperitoneally), and by performing a surgical incision on the perineum, the bulbospongiosus genital muscles were identified and exposed. Two electrodes (EL 452, 12 mm, BIOPAC) were inserted into the bulbospongiosus muscles to record electromyographic (EMG) activity. For a better visualization of the motor genital activity associated with the ejaculation, an additional surgery was performed to expose the bulbar portion of the penis and its anatomical connections with the striated bulbospongiosus muscles. At the end of the surgical approach, the spinal cord was blunt transected around T6 spinal level and prepared for EMG recording. Treatments were administered by infusing the selected compounds into the jugular vein.

Activation and recording of the rhythmic genital motor pattern of ejaculation

Immediately after spinal cord transection, ejaculatory motor pattern was reflexively expressed and recorded in the genital muscles of all animals. To establish the capacity of the spinal apparatus to produce the genital rhythmic pattern after spinalization, two consecutive ejaculatory motor patterns were repeatedly evoked at 3-min intervals by the injection of saline solution (200 μl/min) through a PE-50 catheter (0.965 mm o.d.) inserted into the pelvic urethra through a bladder incision. Injection of saline solution was directed to increase the intraurethral pressure to simulate the urethral distention produced by the emptying of the contents of the accessory glands into the posterior urethra. Thereafter, one of the selected treatments was intravenously applied and the number, frequency of contractions of the striated bulbospongiosus muscles and its latency of response obtained under their influence were recorded for 5 min, which was registered on a polygraph (Biopac Student Lab PRO, version 3.7.3, frequency 50Hz and model MP36E-CE). Five minutes after recording the EMG in each sequential treatment, three consecutive urethral stimulations were monitored at 3 min intervals, as described above. The latency of contractions was expressed as the time elapsed from the application of a test stimulus until the first contraction of the bulbospongiosus muscles. The number of motor contractions included all motor contractions expressed in the motor ejaculatory train evoked by the sensorial or pharmacological stimuli. The frequency of contractions of the bulbospongiosus muscles was calculated by dividing the number of contractions by the duration of the motor train.

Supplementary Table S1: Some pharmacological activities of selected compounds detected in PHF

Name of the compound	Pharmacological activities	References
Hydroquinone	Antimicrobial<br>anti-inflammatory<br>skin lightening<br>Anti-Cancer<br>Pro-apoptotic<br>antioxidant	Ma et al., 2019<br>Chandra et al., 2012<br>Tse et al., 2010<br>Byeon et al., 2018<br>Chen et al., 2004<br>Chepeleva et al., 2021
(-)-Norephedrine	anorexic<br>anti-autistic	Wellman et al., 1990<br>Meistrup-Larsen et al., 1978
Erythritol	antitumor<br>antioxidant<br>Anti-diuretic and anti-alpha-glucosidase	Alamri et al., 2022<br>den Hartog et al., 2010<br>Nelson et al., 2024
Quinic acid	antioxidant and anti-tyrosinase<br>anti-alpha-glucosidase<br>anti-fungal	Choi et al., 2021<br>Chen et al., 2022<br>Ma et al., 2010
Palmitic acid	androgenic<br>antitumor	Baddela et al., 2022<br>Harada et al., 2002
Eicosadienoic acid	anti-inflammatory	Huang et al., 2011
Linoleic acid	antitumor<br>antiapoptotic<br>antioxidant<br>antibacterial	Lauson et al., 2023<br>Lee et al., 2022<br>Xu et al., 2020<br>Kusumah et al., 2020
Oleic acid	anti-Inflammatory<br>antioxidant<br>antibacterial	Santa-María et al., 2023<br>Liu et al., 2023<br>Ramadan et al., 2024

Supplementary Table S2: Total phenolic and flavonoid contents in PHF.

Plant extract	**Total phenolics**<br><br>**(mg of GAE/g DW)**	**Total flavonoids**<br><br>**(mg of QE/g DW)**
**PHF**	150.88 ± 6.51	5.63 ± 0.71

Supplementary Table S3: Pharmacokinetics and ADME properties of compounds identified in PHF.

Name of the compound	Molecular Weight	No. of rotatable bonds	H-Bond (Donor/ Acceptor)	Molar Refractivity	TPSA (Å²)	Log P	GI absorption	BBB permeant	Lipinski's Rules satisfied	Bioavailability Score
Oxirane, (methoxymethyl)	88.11	2	0/2	21.40	21.76	-0.57	High	No	5/5	0.55
p-Menthone	154.25	1	0/1	48.27	17.07	2.30	High	Yes	5/5	0.55
Eucalyptol	154.25	0	0/1	47.12	9.23	2.45	High	Yes	5/5	0.55
Carbamimidic acid	60.06	0	2/1	12.92	69.11	-1.60	High	No	5/5	0.55
Urea, n-methyl-n-nitroso-	103.08	2	1/3	22.66	75.76	-1.12	High	No	5/5	0.55
Cycloglycylalanine	128.13	0	2/2	37.87	58.20	-1.34	Low	No	5/5	0.55
Cyclopentanone dimethylhydrazone	126.2	1	0/1	40.23	15.60	1.23	High	Yes	5/5	0.55
Methanamine, n-(1-methyl-2-pyrrolidinylidene)-	112.17	0	0/1	39.34	15.60	0.88	Low	No	5/5	0.55
2-Heptanamine, 5-methyl-	129.24	4	1/1	43.28	26.02	2.22	High	Yes	5/5	0.55
Maleamide	114.1	2	2/2	26.68	86.18	-1.45	High	No	5/5	0.55
N1,N1-dimethyl-n2-isopropylformamidine	114.19	2	1/2	26.95	29.26	-0.18	Low	No	5/5	0.55
2(3h)-Furanone, 5-methyl-	98.1	2	2/2	26.68	86.18	-1.45	High	No	5/5	0.55
4h-Pyran-4-one, 2,3-dihydro-3,5-dihydroxy-6-methyl-	144.12	0	2/4	32.39	66.76	-1.77	High	No	5/5	0.85
pyrocatechol	110.11	0	2/2	30.49	40.46	0.79	High	Yes	5/5	0.55
N-(2-thienylmethyl)-2-pyridinamine	190.27	3	1/1	55.91	53.16	1.63	High	Yes	5/5	0.55
Hydroquinone	110.11	0	2/2	30.49	40.46	0.79	High	Yes	5/5	0.55
5'-O-[n,n-dimethylsulfamoyl]adenosine	374.38	5	3/10	84.07	174.30	-2.57	Low	No	4/5	0.55
(-)-Norephedrine	151.21	2	2/2	44.89	46.25	1.26	High	Yes	5/5	0.55
Tetrahydro-3,4-furandiol	104.1	0	3/2	22.64	49.69	-1.45	High	No	5/5	0.55
4,5,6,6a-Tetrahydro-2(1h)-pentalenone	122.16	0	0/1	36.07	17.07	1.58	High	Yes	5/5	0.55
Erythritol	122.12	3	4/4	25.99	80.92	-1.91	Low	No	5/5	0.55
Quinic acid	192,17	1	5/6	-2.14	118.22	-2.14	Low	No	5/5	0.56
Alpha-methyl-DL-phenylalanine	179.22	3	2/3	50.35	63.32	-0.81	High	Yes	5/5	0.55
Palmitic acid	256.42	14	1/2	80.80	37.30	4.19	High	Yes	4/5	0.85
Eicosadienoic acid	308.5	16	1/2	99.08	37.30	-	-	-	-	-
Linoleic acid	280.4	14	1/2	89.46	37.30	-	-	-	-	-
Oleic acid	282.5	15	1/2	89.94	37.30	4.57	High	No	4/5	0.85
(Tetrahydroxycyclopentadienone)tricarbonyliron(0)	283.957	4	0/1	124.40	17.07	5.61	Low	No	4/5	0.55
5-Methyl-2(5H)-furanone	98.1	0	0/2	24.85	26.30	0.40	High	No	5/5	0.55
Pulegone	152.23	0	0/1	47.80	17.07	2.20	High	Yes	5/5	0.55

Supplementary Table S4: Molecular docking values of selected compounds from PHF against TRPV1(PDB ID: 5IS0) and TRPM2 (PDB ID: 6PUS)

			TRPV1				TRPM2	
**Compounds**	**Affinity (kcal/mol)**	**Number of H bonds**	**Alkyl/Pi-Alkyl interactions**	**Van-der Walls interactions**	**Affinity (kcal/mol)**	**Number of H bonds**	**Alkyl/Pi-Alkyl interactions**	**Van-der Walls interactions**
p-Menthone	-9.83	0	Leu-1423, Phe-1426, Leu-664, Cys-1422, Met-1392, Ile-668, Leu-1396	Leu-1399, Thr-1426	-9.54	0	Phe-1888, Trp-1895, Val-1486, Phe-1910, Tyr-1881	Leu-1974, Asn-1893, Thr-1410, Trp-1411, Tyr-1409, Asn-1483
Eucalyptol	-8.69	0	Leu-1450, Phe-1748, Ala-1453, Ala-1751, Phe-1402, Met-1752	Ile-1449, Val-1747, Leu-1457, Thr-1755	-9.86	0	Val-534, Tyr-584, Trp-591, Tyr-520, Ile-566	Val-547, Leu-515, Cys-513, Ile-559, Ala-522, Gly-511, Val-512. Ala-521, Val-568
Pyrocatechol	-7.73	2 (Tyr-49, Gln-64)	Pro-336	Gln-333, Phe-337, Leu-307, Val-304, Gly-303, Asp-306, Gln-51	-7.16	1 (Ile-5123)	Ile-3821	Ile-2519, Ile-1148, Ile-2522, Ile-1151, Ser-5127, Lys-5122, Pro-5119
N-(2-thienylmethyl)-2-pyridinamine	-10.02	0	Leu-1941, Ile-1054, Leu-588	Ile-642, Tyr-631, Leu-585, Tyr-584, Met-581, Ile-1062	-9.03	0	Tyr-572	Arg-601, Phe-335
Hydroquinone	-7.99	2 (Asp-576, Asn-1070)	Ile-1066, Leu-678	Met-1071, Met-572, Ile-573, Leu-577, Met-581, Phe-580	-7.27	0	Ile-1151, Ile-2519	Ile-5123, Ile-3821, Ile-3824, Lys-1147, Ser-1152
(-)-Norephedrine	-9.96	1 (Tyr-852)	-	Phe-856, Phe-853, Asn-854, Phe-851, Tyr-972, Gly-975, Phe-976, Arg-974	-8.60	2 (Ile-3821)	Ile-5126, Ile-2519, Ile-2522, Ile-1151, Ile-1148	Lys-3820, Ile-3824, Ser-3825, Lys-5122, Glu-3822
Alpha-methyl-DL-phenylalanine	-8.41	1 (Arg-221)	Tyr-311	Ile-369, Ile-411, Asn-411, Gly-220, Val-218, Tyr-215, Tyr-316	-8.03	1 (Arg-13)	Tyr-572	Ala-556, Gly-571, Tyr-334, Phe-577
Pulegone	-10.06	0	Leu-1399, Leu-664, Tyr-1395, Leu-1396. Met-1392, Ile-668	Leu-1423, Cys-1422	-9.97	0	-	Asn-204, Gly-205, Phe-8, Pro-53, Leu-52, Cys-101, Ile-104, Arg-100, Gly-12, Tyr-108
Capsazepine/ ACA	-11.25	1 (Tyr-2255)	Tyr1727, Tyr-2191, Phe-2187, Leu-2195, Tyr-2250, Leu-2258, Tyr-2191	Leu-1731, Ile-1735, Ile-2263, Asn-2260, Thr-2254, Leu-2253	-14.52	2 (Gly-4369)	Lys-4509, Ala-4513, Ile-4373, Ala-4542, Arg-4368, Ile-4545	Thr-4539, Pro-4538, Leu-4508, Gly-4370, Arg-4654, Asp-4579, Leu-4578, Asn-4577, Lys-4548, Leu-4510

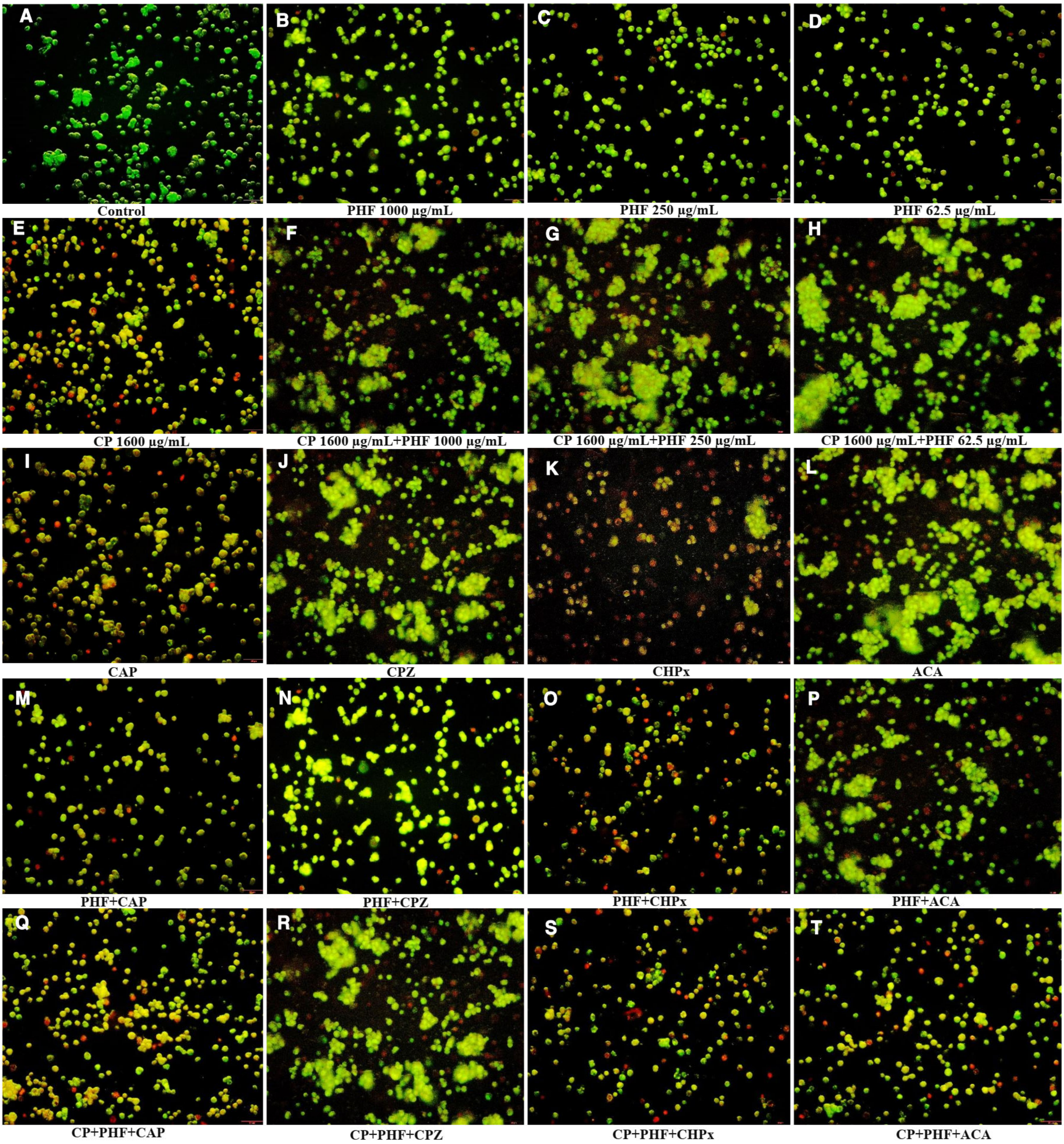

Figure S1: Apoptotic cells detected by AO/EB staining.

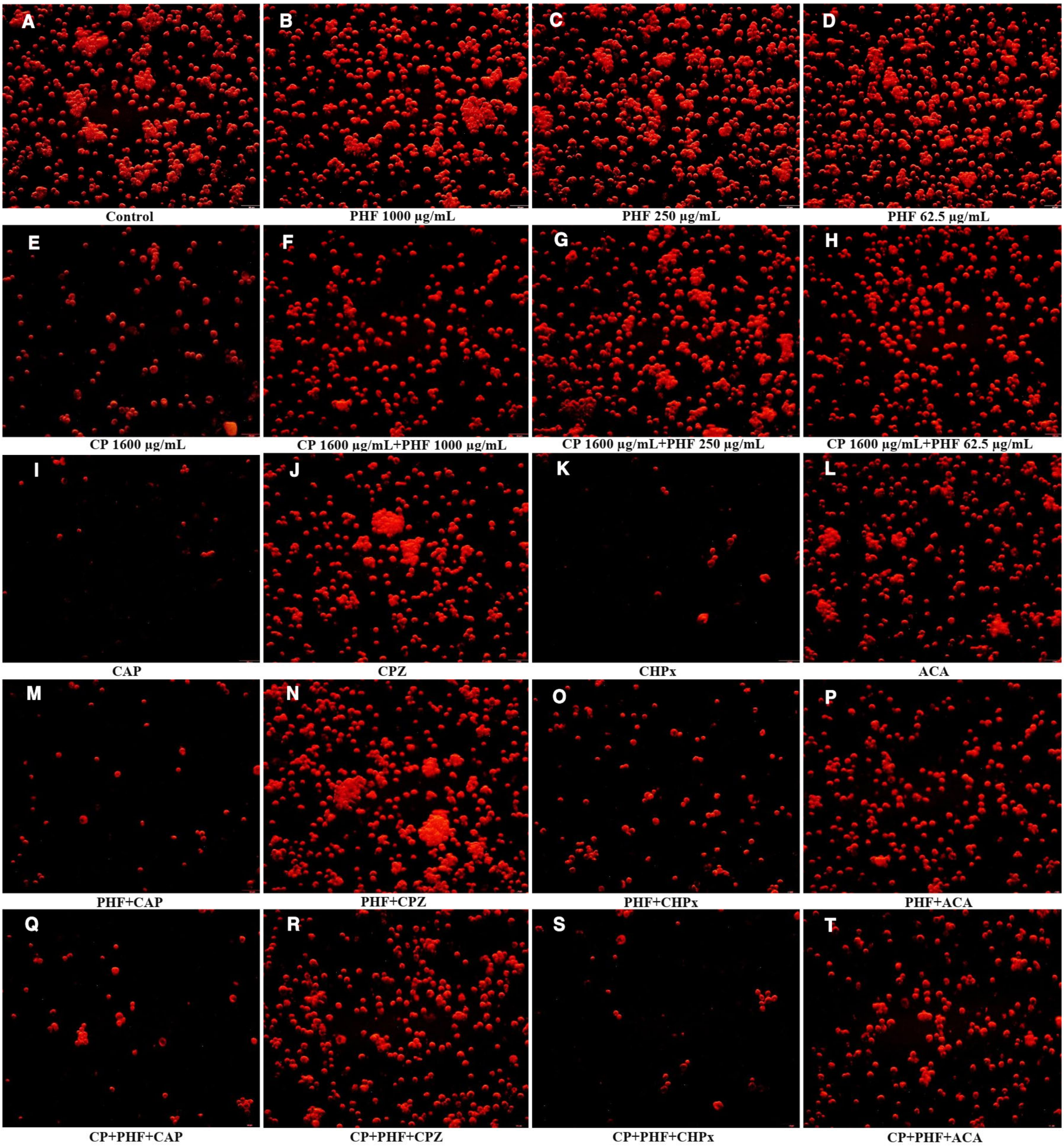

Figure S2: RH123 fluorescent images depicting the effects of various treatments on mitochondrial membrane potential in TM3 cells.

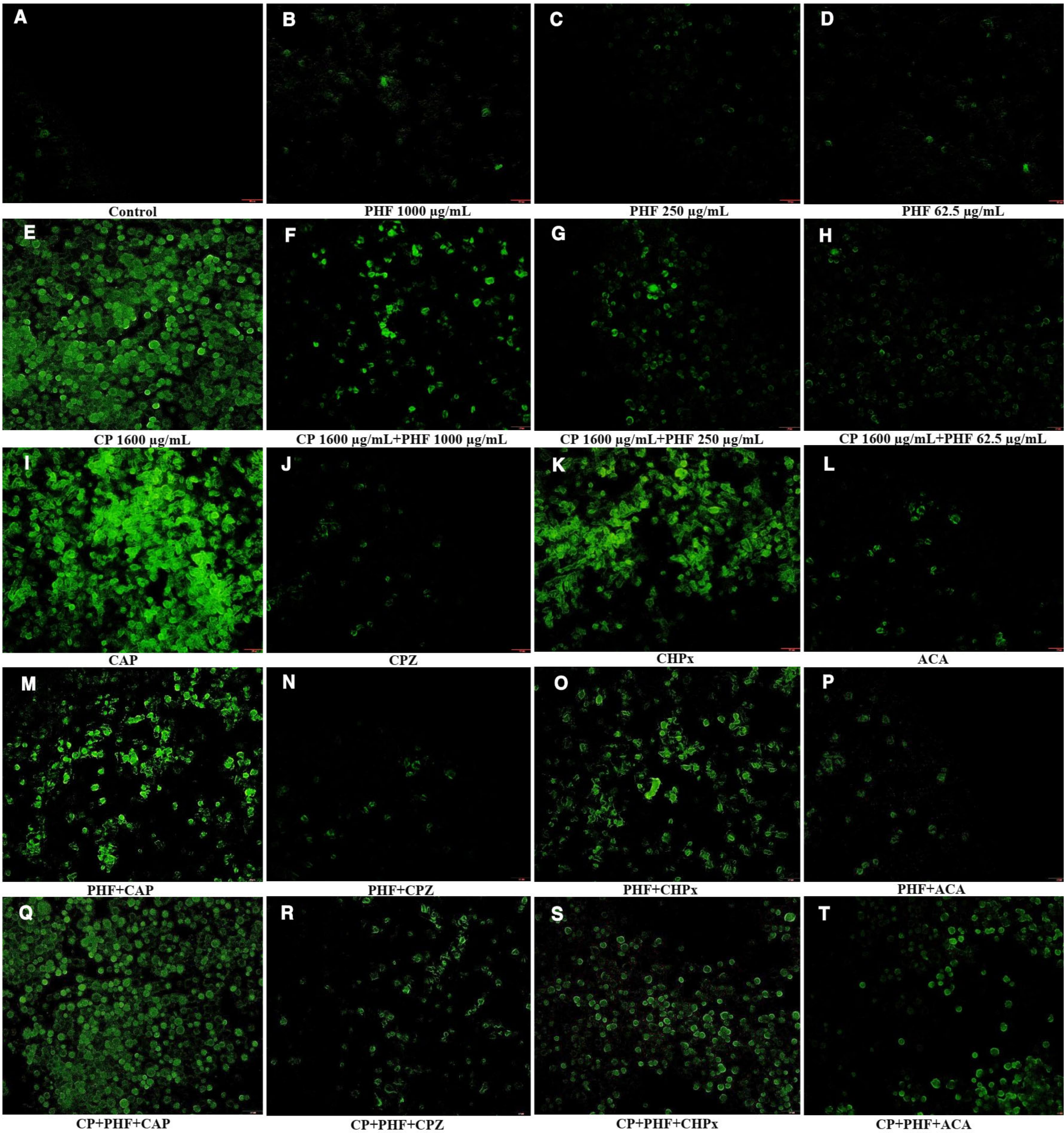

Figure S3: DCF-DA images depicting the effects of various treatments on ROS generation in TM3 cells.

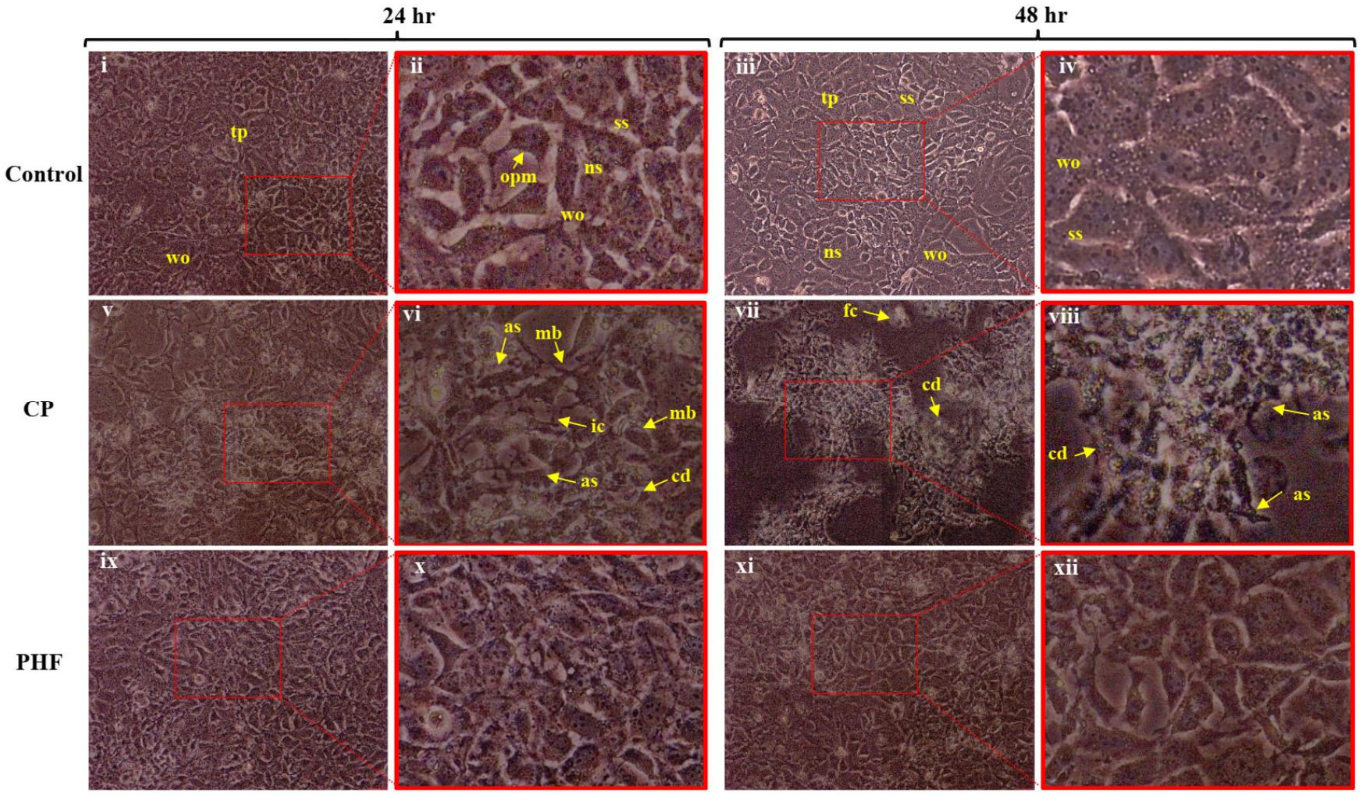

Figure S4: Effects of treatments on cell morphology.
